# How RNA transcripts coordinate DNA recombination and repair

**DOI:** 10.1038/s41467-018-03483-7

**Published:** 2018-03-15

**Authors:** Shane McDevitt, Timur Rusanov, Tatiana Kent, Gurushankar Chandramouly, Richard T. Pomerantz

**Affiliations:** Department of Medical Genetics and Molecular Biochemistry, Fels Institute for Cancer Research, Temple University Lewis Katz School of Medicine, Philadelphia, PA 19140 USA

## Abstract

Genetic studies in yeast indicate that RNA transcripts facilitate homology-directed DNA repair in a manner that is dependent on RAD52. The molecular basis for so-called RNA−DNA repair, however, remains unknown. Using reconstitution assays, we demonstrate that RAD52 directly cooperates with RNA as a sequence-directed ribonucleoprotein complex to promote two related modes of RNA−DNA repair. In a RNA-bridging mechanism, RAD52 assembles recombinant RNA−DNA hybrids that coordinate synapsis and ligation of homologous DNA breaks. In an RNA-templated mechanism, RAD52-mediated RNA−DNA hybrids enable reverse transcription-dependent RNA-to-DNA sequence transfer at DNA breaks that licenses subsequent DNA recombination. Notably, we show that both mechanisms of RNA−DNA repair are promoted by transcription of a homologous DNA template in *trans*. In summary, these data elucidate how RNA transcripts cooperate with RAD52 to coordinate homology-directed DNA recombination and repair in the absence of a DNA donor, and demonstrate a direct role for transcription in RNA−DNA repair.

## Abstract (web summary)

Homologous recombination (HR) typically uses DNA as a donor template to accurately repair DNA breaks. Here, the authors elucidate two mechanisms by which RAD52 uses RNA as a template for HR: one involving RNA-mediated synapsis of a homologous DNA break, and the other involving reverse transcriptase dependent RNA-to-DNA sequence transfer at DNA breaks.

## Introduction

Homologous recombination (HR) utilizes a DNA donor as a template for DNA repair synthesis and is therefore the most accurate form of double-strand break (DSB) repair that is essential for suppressing genome instability and tumorigenesis^1,2^. Whether non-canonical forms of recombinational repair exist and contribute to genome integrity, in particular within expressed genes, is an important area of investigation. For example, recent genetic studies in yeast demonstrate that DSBs can be repaired by RAD52 in a manner that depends on the use of a homologous RNA transcript as a template^3^. Specifically, spliced RNA transcripts were implicated in serving as templates for DNA repair synthesis at homologous DSBs either in* cis* or in* trans* of the transcription site^3^. This form of RNA transcript-dependent DNA recombinational repair (RNA−DNA repair) was shown to be promoted by RAD52 and putative reverse transcriptase (RT) activity, while being suppressed by RNase H^3^. RAD52 was also shown to promote RNA−DNA annealing in vitro^3^. Hence, these studies support RAD52-mediated RNA−DNA hybrid formation in transcript-dependent RNA−DNA repair. Additional studies suggest a conserved role for RAD52 in transcript-dependent DNA repair. For instance, mammalian RAD52 was shown to be preferentially recruited to DSBs in a transcription-dependent manner during G0 and G1 cell-cycle stages^4^, and multiple reports demonstrate that RAD52 associates with RNA polymerase II and RNA transcripts in mammalian cells^5,6^.

**Fig. 1:**
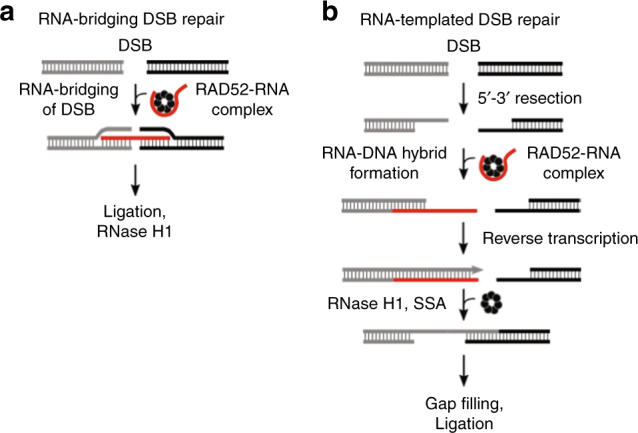
Models of RAD52-mediated RNA−DNA repair. **a** RNA-bridging DSB repair model. RAD52 utilizes RNA to tether both ends of a homologous DSB which forms a DNA synapse for ligation. RNA degradation by RNase H may also occur. **b** RNA-templated DSB repair model. RAD52 forms an RNA−DNA hybrid along the 3′ overhang of a DSB. The RNA is then used as a template for DNA repair synthesis by RT. The RNA is then degraded by RNase H and RAD52 promotes SSA of the opposing homologous ssDNA overhangs. Final processing of the DSB involves gap filling and ligation

Although it is unknown how RAD52 promotes RNA−DNA repair, we envisaged the following two models based in part on yeast genetics^3^. In the first, RAD52 is predicted to promote RNA-bridging of a homologous DSB, thus facilitating sequence-directed DNA synapsis and ligation (Fig. 1a). In the second model, RAD52 is predicted to promote annealing between RNA and the 3′ ssDNA overhang of a resected homologous DSB, forming a RNA−DNA half-bridge (Fig. 1b). The annealed RNA then serves as a template for RT, generating homology for subsequent RAD52 single-strand annealing (SSA) of the opposing 3′ overhang. Here, we fully investigate the molecular basis of RNA–DNA repair involving RAD52 by reconstituting the mechanisms illustrated in Fig. 1. Our data demonstrate that RAD52 cooperates with RNA as a homology-directed ribonucleoprotein complex to facilitate both mechanisms of RNA−DNA repair. We further show that these modes of RNA−DNA repair are promoted by transcription of a homologous DNA template in* trans*. Together, these data reveal how RNA transcripts act with RAD52 to coordinate homology-directed DNA recombination and repair in the absence of a DNA donor, and directly demonstrate an essential function for transcription in RNA−DNA repair.

## Results

### RAD52 promotes RNA-dependent DNA recombination

**Fig. 2:**
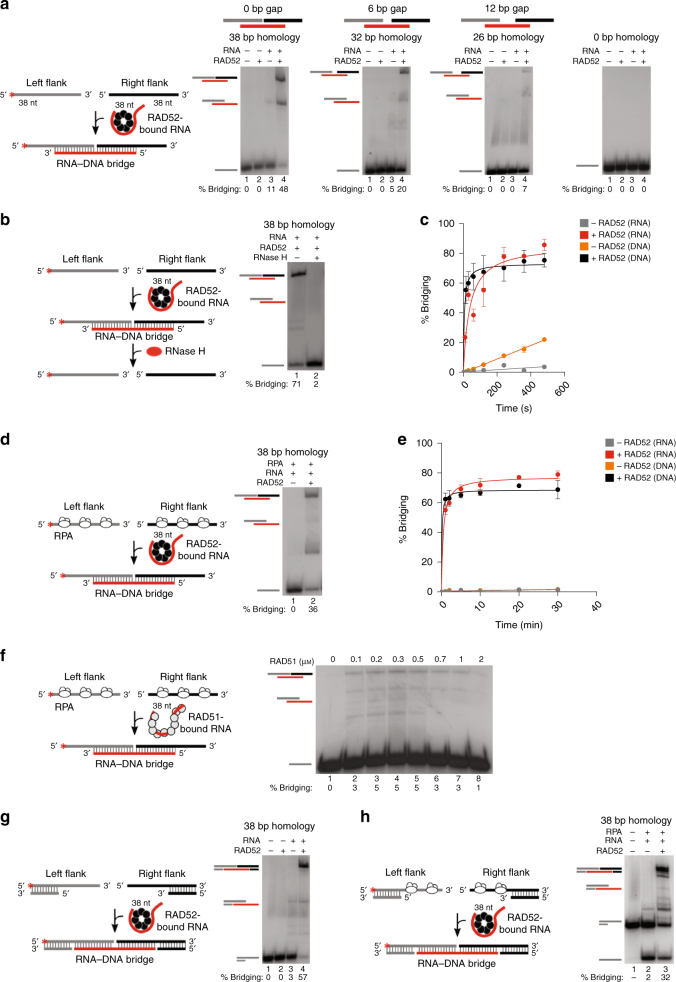
RAD52 promotes RNA-dependent DNA recombination. **a** Schematic of assay (left). Non-denaturing gels showing RAD52 RNA−DNA recombination (RNA-bridging of homologous DNA) in the presence of the indicated substrates (right). **b** Schematic of assay (left). Non-denaturing gel showing RNase H digestion of a RAD52-mediated RNA−DNA recombination intermediate (RNA−DNA recombinant bridge) (right). **c** Graph showing a time course of RNA–DNA recombination (bridging) compared to DNA−DNA recombination (bridging) of left and right flanking ssDNA without RPA and in the presence and absence of RAD52. Data shown as average ± SD, *n* = 3. **d** Schematic of assay (left). Non-denaturing gel showing RAD52 RNA−DNA recombination in the presence of the indicated RPA-coated substrates (right). **e** Graph showing a time course of RNA−DNA recombination (bridging) compared to DNA−DNA recombination (bridging) of left and right flanking RPA-bound ssDNA in the presence and absence of RAD52. Data shown as average ± SD, *n* = 3. **f** Schematic of assay (left). Non-denaturing gel showing RAD51 RNA−DNA recombination (bridging) in the presence of RPA pre-coated substrates (right). **g** Schematic of assay (left). Non-denaturing gel showing RAD52 RNA−DNA recombination (bridging) of the indicated pssDNA substrates (right). **h** Schematic of assay (left). Non-denaturing gel showing RAD52 RNA−DNA recombination (bridging) of the indicated RPA-coated pssDNA substrates (right). ^*^ = ^32^P label. % bridging indicated

To definitively determine a direct role for RNA transcripts in coordinating recombinational repair, we reconstituted the RAD52-mediated RNA−DNA repair mechanisms illustrated in Fig. 1. First, we confirmed that RAD52 exhibits a high affinity for RNA (*K*_d_ = 97 nm) and efficiently promotes RNA−DNA annealing in a similar manner to DNA−DNA annealing, as shown previously (Supplementary Fig. 1A, B)^3^. Consistent with prior reports, RAD52 facilitates DNA−DNA and RNA−DNA annealing in the presence and absence of the ssDNA binding protein RPA^3,7–12^. Next, the ability of RAD52 to promote the RNA-bridging mechanism of RNA−DNA repair was investigated (Fig. 1a). This process would require RAD52 to assemble a RNA−DNA hybrid that spans both ends of the DNA break, resulting in a RNA−DNA recombinant bridge (Fig. 1a). This tri-molecular form of RAD52 annealing, referred to herein as bridging, has not previously been investigated. Initially, we tested whether RAD52 promotes RNA-bridging of various model DNA break substrates. RAD52 was pre-incubated with RNA 38 nucleotides (nt) in length, then mixed with different radio-labeled left and right flanking ssDNA oligonucleotides which respectively share particular lengths of sequence homology at their 3′ and 5′ regions with the RNA (Fig. 2a). Reactions were terminated by the addition of excess unlabeled ssDNA, EDTA, SDS, and proteinase K that degrades protein. Radio-labeled nucleic-acids were resolved in non-denaturing polyacrylamide gels and visualized by autoradiography. Consistent with the RNA-bridging model, RAD52 cooperates with RNA to efficiently combine the left and right DNA flanks that share a total of 38 nt of sequence homology with the RNA (Fig. 2a, left gel). Pre-annealed radio-labeled recombinant RNA−DNA bridges were used as markers (Supplementary Fig. 1C). Reducing the amount of homology between the RNA and left and right DNA flanks to 32 nt and 26 nt resulted in corresponding reductions in RAD52-dependent RNA-bridging (Fig. 2a, middle panels). As expected, RAD52 fails to promote RNA-bridging of non-homologous DNA (Fig. 2a, right panel), and RNA-bridging is not specific to particular sequences (Supplementary Fig. 1D). To further confirm that RAD52 assembles the expected RNA−DNA intermediate, we assessed whether these structures are susceptible to RNase H that degrades the RNA portion of RNA−DNA hybrids. Indeed, RAD52 assembled RNA−DNA bridges are hydrolyzed by RNase H (Fig. 2b). This is consistent with yeast genetics showing that RNA−DNA repair is inhibited by RNase activity^3^. Importantly, we find that this mechanism of RAD52-mediated RNA−DNA bridging occurs under various physiological concentrations of magnesium (Supplementary Fig. 2A). RAD52 can also use ssDNA to assemble recombinant DNA−DNA bridges, and these reactions occur on a similar time scale as those with RNA (Fig. 2c). Minor DNA bridging is observed in the absence of RAD52 due to spontaneous DNA−DNA annealing (Fig. 2c). We observe less spontaneous RNA−DNA annealing compared to DNA−DNA annealing, presumably due to more prominent RNA secondary structure (Supplementary Fig. 1B).

Considering that RAD52 functions with RPA during DNA recombinational repair, we tested whether RAD52 promotes RNA-bridging of the ssDNA flanks pre-coated with RPA (Supplementary Fig. 1E)^13^. The results show that in the presence of RPA, RAD52 promotes RNA-bridging, albeit at a significantly slower rate (Fig. 2d; compare Fig. 2e with Fig. 2c). As a control, we demonstrate that the bridging reaction with RPA occurs at multiple different magnesium concentrations (Supplementary Fig. 2B). RAD52 also functions with ssDNA to facilitate the bridging reaction with RPA present (Fig. 2e). Since RPA suppresses spontaneous annealing, no bridging is observed in the absence of RAD52, as expected (Fig. 2e). Although RAD51 similarly binds to RNA (Supplementary Fig. 1F), it is unable to efficiently promote RNA-bridging like RAD52 (Fig. 2f). Hence, these data along with previous genetic studies indicate a specific function for RAD52 in RNA−DNA repair^3^.

Next, we tested whether RAD52 performs RNA-bridging of a model DSB harboring short ssDNA overhangs, referred to as partial ssDNA (pssDNA) (Fig. 2g). Short ssDNA overhangs can result from so-called “dirty” breaks caused by chemical agents, reactive oxygen, and radiation. Endonuclease activity and limited resection by the Mre11-Rad50-NbsI-CtIP complex can also generate short ssDNA overhangs^14,15^. Similar to the reactions with ssDNA, RAD52 promotes RNA-bridging of homologous pssDNA to facilitate DNA synapse formation (Fig. 2g). RNA-bridging also occurs when the pssDNA is pre-coated with RPA (Fig. 2h; Supplementary Fig. 1E). We note that RPA promotes partial unwinding of the pssDNA substrate (Fig. 2h). RPA DNA unwinding has previously been characterized and confirmed in control experiments (Supplementary Fig. 1G)^16,17^.

### RAD52 promotes RNA transcript-dependent DNA repair

**Fig. 3:**
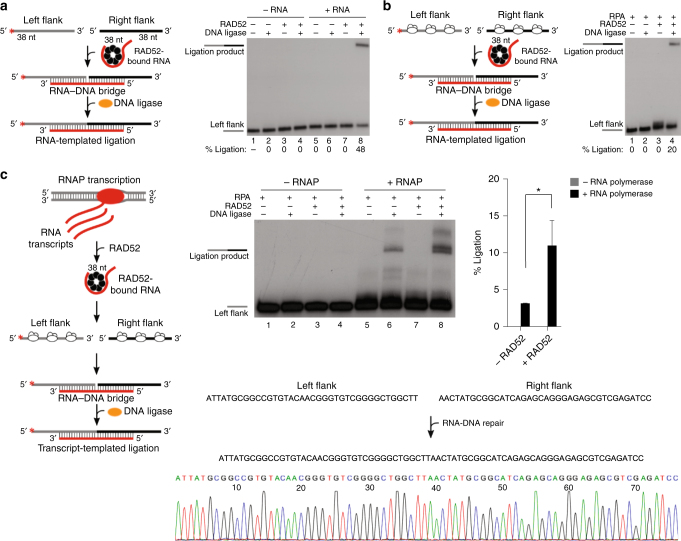
RAD52 promotes RNA transcript-dependent DNA recombinational repair. **a** Schematic of assay (left). Denaturing gel showing RAD52-dependent RNA−DNA repair in the presence of left and right ssDNA flanks and the indicated proteins (right). **b** Schematic of assay (left). Denaturing gel showing RAD52-dependent RNA−DNA repair in the presence of RPA-coated left and right ssDNA flanks and indicated proteins (right). **c** Schematic of assay (left). Denaturing gel showing RAD52-mediated RNA transcript-dependent DNA repair in the presence of RPA-coated left and right ssDNA flanks and indicated proteins (middle). Graph showing percent of RNA transcript-dependent DNA recombinational repair (right). Data shown as average ± SD, *n* = 3. *, *p* = 0.016 (unpaired Student’s *t*-test). Sequencing chromatogram of RNA transcript-dependent DNA recombinational repair product (bottom). ^*^ = ^32^P label

We proceeded to examine whether RAD52 RNA−DNA bridging facilitates DNA ligation which would document an accurate form of RNA-directed DNA recombinational repair (Fig. 3a, left). Indeed, RAD52-dependent RNA−DNA bridging generates DNA synapses that are sealed by DNA ligase in the presence and absence of RPA (Fig. 3a, b). Human DNA ligase 3 and bacteriophage T4 ligase act on these RNA−DNA hybrids (Fig. 3a, b; Supplementary Fig. 3A). Next, we probed whether RAD52 utilizes bona fide RNA transcripts to perform the RNA-bridging mechanism of RNA−DNA repair. Here, T7 RNA polymerase (RNAP) transcription in* trans* was used to generate homologous RNA transcripts during the RNA−DNA repair reaction (Supplementary Fig. 3B). Transcription was initiated followed by the addition of RAD52, left and right RPA-coated DNA flanks, and finally ligase (Fig. 3c, left). The reactions were terminated and resolved in denaturing gels alongside markers. While transcripts give rise to some repair products in the absence of RAD52, presumably due to the abundance of RNA generated by RNAP, RAD52 clearly enhances transcription-dependent RNA−DNA repair (Fig. 3c, right). Sequence analysis of the transcription-dependent RNA−DNA repair products confirms the illustrated mechanism of RNA transcript-dependent recombinational repair (Fig. 3c, bottom). These data demonstrate a RNA-bridging mechanism of RNA−DNA repair that can conceivably help preserve genetic integrity in transcribed regions^3^.

### RAD52 promotes DSB repair via RNA−DNA bridging

**Fig. 4:**
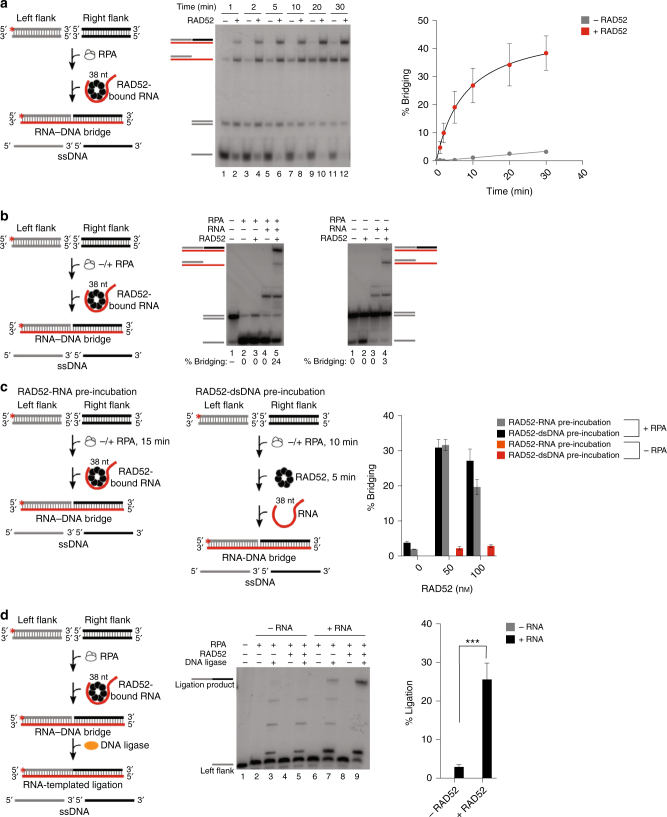
RAD52 promotes RNA-dependent recombinational repair of DSBs. **a** Schematic of assay (left). Non-denaturing gel showing a time course of RAD52-dependent RNA−DNA recombination (bridging) of blunt-ended DNA in the presence of RPA (middle). Plot showing time course of RAD52-dependent RNA−DNA recombination (bridging) of blunt-ended DNA in the presence of RPA (right). Data shown as average ± SEM, *n* = 3. **b** Schematic of assay (left). Non-denaturing gels showing RAD52-dependent RNA−DNA recombination (bridging) of blunt-ended DNA in the presence (left) and absence (right) of RPA. **c** Schematic of assays showing RAD52-dependent RNA−DNA recombination (bridging) of blunt-ended DNA employing either RAD52-dsDNA pre-incubation (right schematic) or RAD52-RNA (left schematic) pre-incubation steps, and performed either with and without RPA. Graph showing quantification of RAD52-dependent RNA−DNA recombination (bridging) of blunt-ended DNA utilizing the indicated pre-incubation steps and with and without RPA (right). Data shown as average ± SD, *n* = 3. **d** Schematic of assay (left). Denaturing gel showing RAD52-dependent RNA−DNA recombinational repair (bridging followed by ligation) of blunt-ended DNA in the presence of the indicated proteins and substrates (middle). Graph showing percent of RAD52-dependent RNA-mediated recombinational repair of blunt-ended DNA (% ligation) (right). Data shown as average ± SD, *n* = 3. ***, *p* = 0.0008 (unpaired Student’s *t-*test). ^*^ = ^32^P label

We proceeded to examine whether this mechanism of recombinational repair involving RNA−DNA recombinant bridges can facilitate DSB repair as modeled in Fig. 1a. For example, we tested whether RAD52 promotes RNA-bridging of a model DSB containing blunt ends, which is often referred to as a “clean” break and represents a physiologically relevant substrate. Here, the reaction conditions used for pssDNA with RPA were repeated with blunt-ended substrates (Fig. 2h, left). Remarkably, the time course demonstrates that RAD52 efficiently promotes RNA-bridging of blunt-ended DNA as indicated by the appearance of the top band (Fig. 4a). These data additionally show that an RNA−DNA half-bridge occurs with increasing time in the absence of RAD52, indicating that RNA−DNA hybrids slowly and spontaneously form in the presence of RPA (Fig. 4a, lanes 7, 9, and 11). Control experiments show that RNA-bridging of the DSB requires RNA and RAD52 (Fig. 4b, left panel), and this reaction also critically depends on RPA (Fig. 4b, right panel; Fig. 4c). Additional controls show that the occasional appearance of intermediate molecular weight byproducts, such as those in Fig. 4b, can form due to RNase contamination and are suppressed by the addition of RNase inhibitors in our reactions (Supplementary Fig. 4A). RPA likely stimulates RNA-bridging of duplex DNA by facilitating DNA unwinding (see lower bands in Fig. 4a, b (left panel), Fig. 2h, and Supplementary Fig. 1G)^16,17^. As a control, we show that RAD52 RNA-bridging of a DSB is compatible with physiologically relevant concentrations of magnesium (Supplementary Fig. 2C).

Recent studies suggest RAD52 reverse strand exchange activity may play a role in RNA−DNA repair^12^. For example, RAD52 pre-bound to double-strand DNA with ssDNA overhangs (i.e. pssDNA) was shown to more efficiently promote strand exchange between RNA and pssDNA compared to reactions performed with RAD52 pre-bound to RNA^12^. We assessed whether pre-incubation of RAD52 with duplex DNA vs. RNA affected the efficiency of our RNA−DNA repair reactions using the model DSB substrate in Fig. 4c. In the absence of RPA, little or no RNA−DNA bridging is observed regardless of whether RAD52 is pre-incubated with DNA or RNA; these data are consistent with a necessary role for RPA in RNA-bridging of blunt-ended DNA (see Fig. 4b, c, Supplementary Fig. 4B). In the presence of RPA, we observe robust RNA-bridging of the DSB in a manner that is dependent on RAD52 as shown in Fig. 4b (Fig. 4c; Supplementary Fig. 4B, bottom right). Altering RAD52 pre-incubation conditions, for example with DNA vs. RNA, has little or no effect on the efficiency of RNA-bridging reactions (Fig. 4c, right; Supplementary Fig. 4B). In the absence of RPA, we find that RAD52-DNA pre-incubation exclusively promotes half bridge RNA−DNA formation with little or no stimulation of full RNA−DNA bridge formation (Supplementary Fig. 4B, bottom left). These data demonstrate that reverse strand exchange can occur in our assays, but does so only in the absence of RPA which does not support full RNA−DNA bridging of a DSB.

We next examined whether RAD52-dependent RNA-bridging of a DSB can serve as a recombination intermediate for ligation. Here, we repeated the conditions used in Fig. 4a; however, ligase was added following the RNA−DNA bridging step and reactions were analyzed in a denaturing urea gel in order to detect the expected ligation product. Remarkably, the data demonstrate that RAD52 acts with RNA and ligase to coordinate recombinational repair of a model DSB as indicated by the identical mobility of the top band with the DNA marker corresponding to the ligation product (Fig. 4d). Controls show that the ligated DSB repair product is undetectable in the absence of RNA, and is greatly diminished when RAD52 is withheld from the reaction (Fig. 4d). Taken together, these data demonstrate a mechanism by which RAD52-RNA complexes promote the repair of blunt-ended DSBs in a homology-directed manner.

### RAD52 promotes RNA transcript-templated DNA recombination

**Fig. 5:**
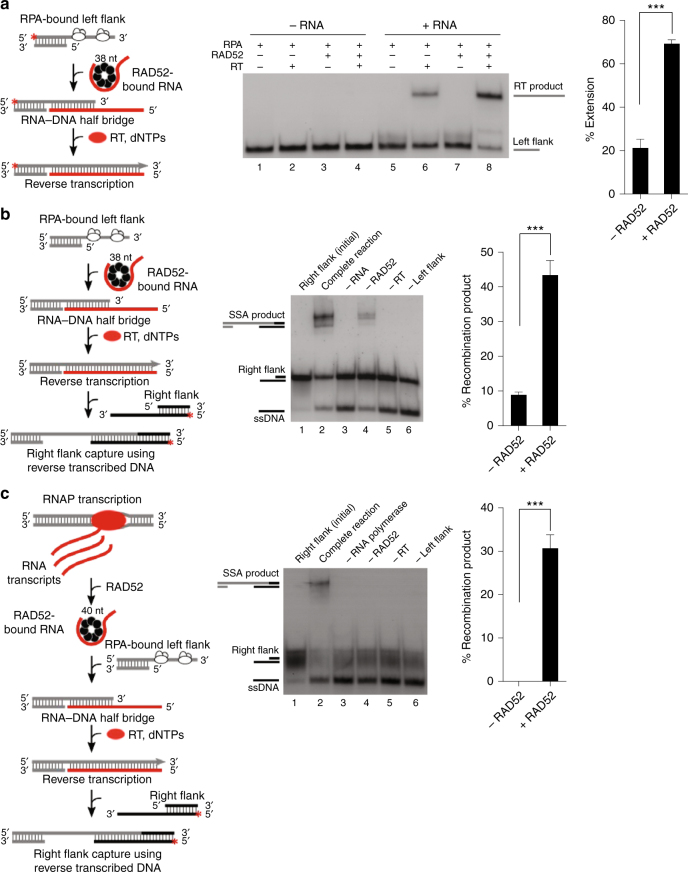
RAD52 promotes RNA transcript-templated DNA recombination. **a** Schematic of assay (left). Denaturing gel showing reverse transcription of a RNA−DNA recombinant half-bridge in the presence of the indicated proteins and RNA (middle). Graph showing percent extension of a RNA−DNA recombinant half-bridge by RT in the presence and absence of RAD52 (right). Data shown as average ± SD, *n* = 4, ***, *p* < 0.001 (unpaired Student’s *t-*test). **b** Schematic of assay (left). Non-denaturing gel showing RNA-templated DNA recombination in the presence of indicated proteins and RNA and DNA substrates (middle). Graph showing percent of RNA−DNA recombination product in the presence and absence of RAD52 (right). Data shown as average ± SD, *n* = 4, ***, *p* < 0.001 (unpaired Student’s *t*-test). **c** Schematic of assay (left). Non-denaturing gel showing RAD52-dependent RNA transcript-templated DNA recombination in the presence of the indicated proteins and DNA substrates (middle). Graph showing percent of RNA transcript-templated DNA recombination product in the presence and absence of RAD52 (right). Data shown as average ±SD, *n* = 3, ***, *p < *0.001, (unpaired Student’s *t*-test). ^*^ = ^32^P label

Next, we tested whether RAD52 promotes a mechanism of RNA−DNA repair compatible with the model in Fig. 1b, which relies on RT activity. Several studies have implicated putative RT activity in RNA-templated DNA repair indicated by the transfer of RNA sequence information to DNA during DNA repair^3,5,18–20^. However, the RTs involved in this form of repair remain to be elucidated. First, it was tested whether RAD52 promotes the formation of a recombinant RNA−DNA half-bridge on a RPA-coated model DSB end harboring a 3′ overhang (left flank), and whether the overhang can then be extended by RT in the presence of deoxy-ribonucleoside triphosphates (dNTPs) and template RNA (Fig. 5a, left). As predicted by the model in Fig. 1b, extension of the left flank depends upon homologous RNA and RT, and is significantly stimulated by RAD52 (Fig. 5a, middle and right).

We further tested the model in Fig. 1b by fully reconstituting RNA-templated repair of a model DSB bearing 3′ overhangs (Fig. 5b, left). The RPA-coated left flank was mixed with RAD52 and homologous RNA, enabling formation of a RNA−DNA half-bridge. Next, the 3′ overhang was extended by the addition of RT along with dNTPs as in Fig. 5a. Finally, the opposing end of the model DSB containing a 3′ overhang (right flank) was added, allowing for RAD52 to recombine the two flanks via SSA which requires transfer of the RNA sequence information to the left DNA flank via RT. The results show that this process is dependent on RNA, RAD52, RT, and both DNA flanks, which supports the model depicted in Fig. 1b and previous genetic studies (Fig. 5b, middle and right)^3^. As further support for RNA transcript-dependent DNA recombinational repair, we probed whether this process is promoted by transcription rather than synthetic RNA. Here, we again performed transcription in* trans* to generate homologous RNA transcripts during the reaction (Fig. 5c, left; Supplementary Fig. 3B). Remarkably, the data clearly demonstrate that recombination of the model DSB requires RNAP, RT, and RAD52, and thus elucidate a templating mechanism by which RNA transcripts coordinate recombination of homologous DSBs in the absence of a DNA donor (Fig. 5c, middle and right).

## Discussion

Although previous genetics supported a role for RNA transcripts in promoting DNA recombinational repair along with RAD52, how RNA and RAD52 functioned in this process remained unclear^3^. Our data demonstrate that RAD52 cooperates with transcript RNA as a ribonucleoprotein complex that coordinates homology-directed DNA recombination and repair via two related mechanisms. In the first mechanism, RAD52 promotes annealing of a single RNA transcript to two ends of a homologous DNA break, forming a recombinant RNA−DNA bridge that acts to tether the break ends and facilitate DNA synapsis and subsequent ligation (Fig. 1a). We show that this mechanism occurs with duplex DNA containing either blunt ends or ssDNA overhangs. In the second mechanism, RAD52 promotes annealing of a RNA transcript to a homologous 3′ overhang of a resected DSB, generating an RNA−DNA half-bridge (Fig. 1b). The RNA transcript is then used as a template for reverse transcription, which generates the necessary DNA homology for second end capture of the opposing 3′ overhang via RAD52 SSA (Fig. 1b). Gap filling and ligation are likely required for final processing of the recombination intermediate.

Our studies also directly demonstrate an essential function for transcription in homology-directed RNA−DNA repair. For example, although previous cellular studies in yeast indicated a role for transcription in DNA recombination, it remained unclear whether the transcription machinery directly contributed to the repair process, or simply generated the necessary transcripts for RNA−DNA repair. In this report, we reconstituted mechanisms of RNA−DNA repair involving transcription of a homologous DNA template in* trans* to the DNA break. In this mode, the transcription complex promotes homology-directed DNA repair by synthesizing the necessary homologous transcript in* trans* which then forms an active ribonucleoprotein complex with RAD52. Once formed, the sequence-directed RNA-RAD52 complex searches for homologous DNA breaks to act upon and then assembles the necessary recombinant RNA−DNA hybrids that enable downstream RNA−DNA repair events.

Importantly, our data demonstrate that the initially formed RAD52-RNA ribonucleoprotein complex facilitates homology search and subsequent RNA-mediated DSB repair by a novel RNA–DNA bridging mechanism (Fig. 1a). In a recent study, RAD52 was shown to promote efficient RNA−DNA recombination in vitro via reverse strand exchange whereby it first forms a complex with DNA and then performs DNA−RNA strand exchange^12^. Although we confirmed the ability of RAD52 to promote reverse strand exchange between double-stranded DNA and RNA, this mechanism did not support actual RNA-mediated DSB repair in our assays. Instead, we found that reverse strand exchange was limited to promoting RNA−DNA recombination at a single DNA end and thus failed to facilitate RNA-mediated DNA synapsis and ligation as illustrated in Fig. 1a. Despite these findings, our data do not rule out a potential role for RAD52 reverse strand exchange in RNA-templated DSB repair via reverse transcription and subsequent SSA (Fig. 1b).

We note that particular reactions involving transcription in* trans* as a source of homologous RNA resulted in relatively low yields of RNA−DNA recombinational repair. Previous studies in yeast demonstrated that RNA−DNA repair is substantially more efficient when transcription occurs in* cis* to the DNA break rather than *in trans*^3^. Thus, close proximity (i.e. in* cis*) of the transcription bubble to the DNA break may provide a mechanistic advantage for RNA−DNA repair. For example, transcription complex unwinding of DNA near the break could conceivably facilitate RNA−DNA annealing by RAD52. In contrast, transcription in* trans* of the DNA break likely has no direct mechanistic link to the repair process aside from generating the necessary homologous transcript.

An important consideration is whether RNA-mediated DNA repair is widespread and important for particular aspects of genome maintenance or plasticity. Multiple lines of research in yeast and mammalian cells support the use of RNA in DNA repair, suggesting an evolutionary advantage for this non-canonical form of genome repair^3–5,19–23^. Although this type of repair process may contribute to genome integrity in transcribed regions, it may alternatively facilitate genomic plasticity by enabling promiscuous RNA-to-DNA sequence transfer events at DSB junctions harboring little or no microhomologous sequence to the RNA. Considering that our data demonstrate that RAD52 requires relatively long tracts of homology to assemble recombinant RNA−DNA hybrids that direct accurate DNA recombination and repair, they suggest RAD52-mediated RNA−DNA repair might contribute to genome integrity such as in transcribed regions. Consistent with this idea, mammalian RAD52 is recruited to DSBs in a transcription-dependent manner, and associates with RNA polymerase II and RNA transcripts^4,5^. Future studies are needed to determine whether the transcription-associated functions of RAD52 are conserved and involved in genome maintenance specifically within transcribed regions.

## Methods

### RNA-dependent DNA recombination of ssDNA and pssDNA

Reactions were performed in 20 μl volume of buffer A (25 mm Tris-HCl pH 7.5, 1 mm DTT, 10 mm NaCl, 0.01% NP-40, 0.1 mg/ml BSA, 10% glycerol) at 37 °C. Various concentrations of MgCl_2_ were used as follows: Fig. 2a, b, d, g, h: 0.5 mm MgCl_2_; Fig. 2c, e, f: 2 mm MgCl_2_. Twenty units RNase inhibitor (Ambion) was added to reactions in Fig. 2c, e. RAD52 (30 nm) was pre-incubated with 4 nm RNA (SM44R) for 5 min. Next, equimolar concentrations of indicated 5′-^32^P-radiolabeled ssDNA or pssDNA (4 nm) were added. Reactions were terminated after 1 min or indicated times by the addition 0.4 μm cold ssDNA, then treated with 2 μl of stop buffer (5 mg/ml proteinase K, 100 mm Tris-HCl pH 7.5, 1.5% SDS, 100 mm EDTA) for 15 min. Nucleic acids were resolved in 15% non-denaturing polyacrylamide gels and visualized by autoradiography. Reactions containing RAD51 included 1 mm ATP and 4 mm CaCl_2_ (Fig. 2f). The time course in Fig. 2c was performed with 30 nm RAD52. For reactions containing RPA (Fig. 2d, e, g, h), 150–200 nm RPA and 40 nm ssDNA or pssDNA templates were pre-incubated in buffer A for at least 15 min at 37 °C. RPA and ssDNA/pssDNA were used at final concentrations of 15–20 and 4 nm, respectively. RAD52 (75  nm) was pre-incubated with 4 nm RNA for 5 min, then was combined with RPA-coated ssDNA/pssDNA substrates. Bridging was carried out for 30 min, then terminated by the addition of 0.4 μm cold ssDNA, and then treated with 2 μl of stop buffer (5 mg/ml proteinase K, 100 mm Tris-HCl pH 7.5, 1.5% SDS, 100 mm EDTA) for 15 min. Nucleic acids were resolved in 15% non-denaturing polyacrylamide gels and visualized by autoradiography. The time course in Fig. 2e (containing RPA) was performed with 50 nm RAD52. ssDNA substrates: 38 bp homology: SM99, SM100, SM44R; 32 bp homology: SM97, SM98, SM44R; 26 bp homology: SM101, SM102, SM44R. pssDNA substrates: SM100/SM16, SM99/SM14.

### RNA-dependent DNA recombination of dsDNA

Reactions were performed in 20 μl volume of buffer A (25 mm Tris-HCl pH 7.5, 1 mm DTT, 10 mm NaCl, 0.01% NP-40, 0.1 mg/ml BSA, 10% glycerol) at 37 °C. Various concentrations of MgCl_2_ were used as follows: Fig. 4b: 0.5 mm MgCl_2_, Fig. 4a, c: 2 mm MgCl_2_. All dsDNA bridging reactions contained 20 units RNase inhibitor (Ambion) except for Fig. 4b. RPA (200  nm) was pre-incubated with left and right flanking dsDNA (40 nm each) for 15 min and was used in the reaction at final concentrations of 20 and 4 nm, respectively. RAD52 (75 nm) was pre-incubated with 4 nm RNA for 5 min, then was combined with RPA-dsDNA pre-incubation reactions, and bridging was carried out for 30 min. Reactions were terminated by the addition of 0.4 μm cold ssDNA, then treated with 2 μl of stop buffer (5 mg/ml proteinase K, 100 mm Tris-HCl pH 7.5, 1.5% SDS, 100 mm EDTA) for 15 min. dsDNA substrates: SM142/SM142C, SM143/SM143C, SM44R.

### RNA-dependent DNA recombinational repair

RAD52-dependent RNA bridging reactions were performed in 20 μl of buffer A as described above (Fig. 2a), followed by ligation with 0.846 μm bacteriophage T4 DNA ligase (New England Biolabs) with 0.5 mm MgCl_2_ (Fig. 4a) for 2 h at 25 °C. Reactions were supplemented with 1 mm ATP during ligation. Reactions were terminated by the addition of stop buffer (45% formamide, 20 mm EDTA). Reactions were resolved in 12% denaturing (urea) polyacrylamide gels and visualized by autoradiography. In the case of RNA-templated ligation of RPA-coated substrates (Fig. 3b), reactions were performed in 20 μl of buffer A as described above (Fig. 2d) and contained 0.5 mm MgCl_2_. In the case of dsDNA ligation (Fig. 4d), RAD52-dependent RNA bridging reactions were performed in 20 μl of buffer A as described above (Fig. 4a), contained 2 mm MgCl_2_, and Ambion RNase inhibitor. Reactions were supplemented with 1 mm ATP during ligation. Reactions were terminated by the addition of stop buffer (45% formamide, 20 mm EDTA). Reactions were resolved in 12% denaturing (urea) polyacrylamide gels and visualized by autoradiography.

### RNA transcript-dependent DNA recombinational repair

Fig. 3c: Reactions were performed in 20 μl of buffer B (25 mm Tris-HCl pH 7.5, 1 mm DTT, 10 mm NaCl, 0.01% NP-40, 6 mm MgCl_2_, 0.1 mg/ml BSA, 10% glycerol) with RNase inhibitor (Ambion) at 37 °C. Reactions contained 448 μm GTP, and 150 μm ATP, CTP, UTP. Transcription was initiated on 2 nm of a dsDNA template (SM85/SM85C) containing a T7 RNAP promoter by the addition of 10 nm T7 RNAP for 10 min at 37 °C. Next, 200 nm RAD52 was added for 5 min, followed by the addition of RPA-coated 5′-^32^P labeled 4 nm ssDNA template (SM100/SM99P). After 10 min, reactions were supplemented with 0.4 μm cold left flanking ssDNA. Ligation was then performed at 25 °C for 2 h in the presence of 0.846 μm T4 DNA ligase and an additional 1 mm of ATP. Reactions were then terminated by the addition of stop buffer (45% formamide, 20 mm EDTA), and resolved in 15% denaturing (urea) polyacrylamide gels, and visualized by autoradiography.

### RNA transcript-templated DNA recombination

Reactions were performed in 20 μl of buffer A containing 2 mm MgCl_2_, 10 μm dNTPs and 20 units RNase inhibitor (Ambion) at 37 °C. RAD52 (100 nm) was pre-incubated with RNA (SM44R) for 5 min. RPA (20  nm) pre-bound to 4 nm of left flanking pssDNA (SM100/SM16) was added and RNA−DNA recombination (RNA-bridging) was performed for 30 min. Next, ten units of AMV-RT (New England Biolabs) was added for 1 h and MgCl_2_ was supplemented to 4 mm (Fig. 5a). Next, 6 nm of the opposing right flanking 5′-^32^P-labeled pssDNA (SM129/SM130) was added and annealing was performed for 30 min (Fig. 5b). In the case of co-transcriptional RNA−DNA recombination (Fig. 5c), reactions were performed in 20 μl of buffer B and included 300 μm GTP, 100 μm ATP, CTP, and UTP, 100 µm dNTPs, 6 mm MgCl_2_, and 20 units RNase inhibitor (Ambion). Transcription was initiated using 4 nm of a dsDNA template (SM85/SM85C) containing a T7 RNAP promoter by the addition of 10 nm T7 RNAP at 37 °C. After 2 min, 200 nm RAD52 was added for 5 min, followed by the addition of 8 nm RPA-coated left flanking pssDNA template (SM100/SM16). Annealing between transcript RNA and pssDNA was carried out for 30 min. Next, ten units of AMV-RT (New England Biolabs) was added for 1 h to allow for reverse transcription to occur. Finally, 6 nm of 5′-^32^P-labeled right flanking pssDNA (SM83/SM84) was added and annealing was performed for 30 min. In Fig. 5b, c, reactions were terminated by the addition of stop buffer (0.83 mg/ml proteinase K, 8.3 mm Tris-HCl pH 7.5, 0.125% SDS, 8.3 mm EDTA) for 15 min at 37 °C. Nucleic acids were resolved in 15% non-denaturing polyacrylamide gels and visualized by autoradiography. In the case of Fig. 5a, reactions were stopped after AMV-RT extension by addition of the following stop buffer: 45% formamide, 20 mm EDTA, then resolved in 15% denaturing (urea) polyacrylamide gels, and visualized by autoradiography.

### DNA and RNA

DNA and RNA was 5′-^32^P labeled with T4 polynucleotide kinase (New England Biolabs) and [γ-^32^P]ATP (PerkinElmer).

RNA−DNA recombination (RNA-bridging) templates: SM83/SM84 (Fig. 5c), SM99/SM14 (Fig. 2g, h), SM100/SM16 (Figs. 2g, h, 5a−c) SM129/SM130 (Fig. 5b), SM142/SM142C (Fig. 4a−d), SM143/SM143C (Fig. 4a−d).

Transcription template: SM85/SM85C (Figs. 3c, 5c).

RNA-DNA recombinational repair templates: SM100 + SM99 (Fig. 3a−c), SM142/SM142C + SM143/SM143C (Fig. 4d).

DNA and RNA oligonucleotides (Integrated DNA Technologies) are as follows (5′−3′). SM14, GGATCTCGACGCTCTCCCT; SM16, TTGTACACGGCCGCATAAT; SM44R, GCUCUGAUGCCGCAUAGUUAAGCCAGCCCCGACACCCG;

SM44C, CGGGTGTCGGGGCTGGCTTAACTATGCGGCATCAGAGC; SM46R, GAAGCAUUUAUCAGGGUUAUUGUCUCAUGAGCGGAUACAUAUUUGAAU; SM51R, /56-FAM (fluorescein)/UUUUUUUUUUUUUUUUUUUUUUUUUUUUU; SM83, GCAAGCTTATGCACGGGGCTCTGATGCCGCATAGTT; SM84, /5Phos/CGTGCATAAGCTTGC; SM85, ATCGATATTAATACGACTCACTATAGGGCTCTGATGCCGCATAGTTAAGCCAGCCCCGACACCCG; SM85C, CGGGTGTCGGGGCTGGCTTAACTATGCGGCATCAGAGCCCTATAGTGAGTCGTATTAATATCGAT; SM97, GCGGCATCAGAGCAGGGAGAGCGTCGAGATCC; SM98, ATTATGCGGCCGTGTACAACGGGTGTCGGGGC; SM99, /Phos/AACTATGCGGCATCAGAGCAGGGAGAGCGTCGAGATCC; SM100, ATTATGCGGCCGTGTACAACGGGTGTCGGGGCTGGCTT; SM101, /Phos/TATGCGGCATCAGAGCAGGGAGAGCGTCGAGATCC; SM102, ATTATGCGGCCGTGTACAACGGGTGTCGGGGCTGG; SM104P, /5Phos/AGACAATAACCCTGATAAATGCTTCAATTCATGTCCAGCCCAAACTCTG; SM105, AACCAATGGACCATTTAACCAATATTCAAATATGTATCCGCTCATG; SM129, GCAAGCTTATGCAGACGGCTCTGATGCCGCATAGTT; SM130, /Phos/CGTCTGCATAAGCTTGC; SM142, AAGCCAGCCCCGAC; SM142C, GTCGGGGCTGGCTT; SM132, AAATAAACATAAAGTAAGTAAGTATAAGGATAATACACAATAAGTAAATGAATAGACATAGAAAATAAAGTAAATTATATAAA; SM133, TTTATATAATTTACTTTATTTTCTATGTCTATTCATTTACTTATTGTGTATTATCCTTATACTTACTTACTTTATGTTTATTT; SM143, CTGCTCTGATGCCGCATAGTT; SM143C, /5Phos/AACTATGCGGCATCAGAGCAG

### Proteins

RAD52, RPA, T7 RNAP, and Lig3 were purified using previously optimized methods^24–27^. T4 DNA ligase was purchased from New England Biolabs.

### Uncropped images

All uncropped gels from the main text are available as Supplementary Fig. 5.

### Data availability

The data that support the findings of this study are available from the corresponding author upon request.

## Electronic supplementary material

Supplementary Information

Peer Review File
