# Supplementary material for: How RNA transcripts coordinate DNA recombination and repair
Source: Nat Commun. 2018 Mar 15;9:1091. doi: 10.1038/s41467-018-03483-7 (PMC5854605; doi:10.1038/s41467-018-03483-7)
Supplement: Supplementary file 1 — Supplementary Information [file 41467_2018_3483_MOESM1_ESM.pdf]

Supplementary Information

How RNA Transcripts Coordinate DNA Recombination and Repair

McDevitt *et. al.*

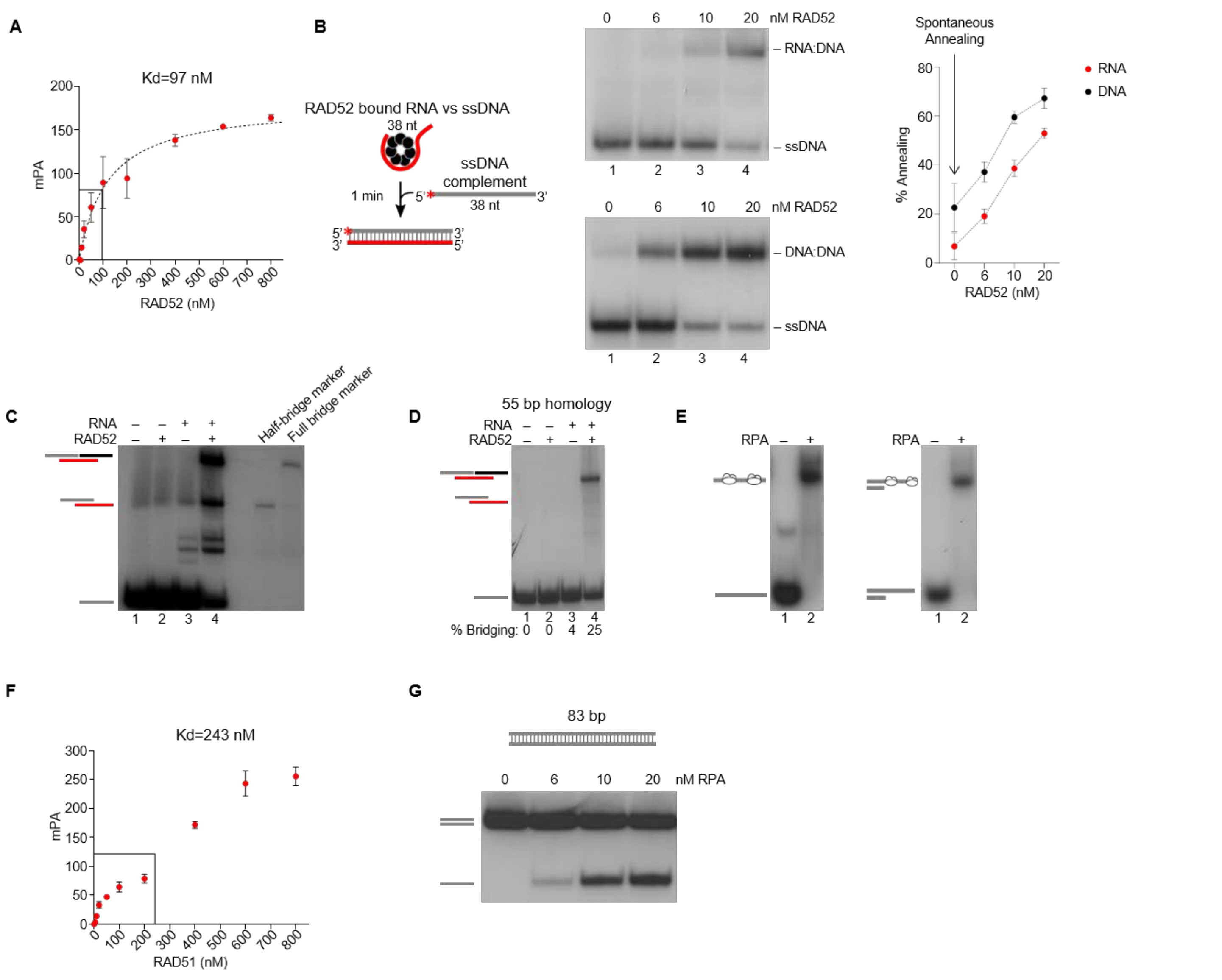

Supplementary Fig. 1 Controls for RAD52-dependent RNA-DNA recombination.

(**A**) Plot showing RAD52 binding to fluorescein-conjugated RNA measured by fluorescence polarization. Data shown as average ± SD, n=3. (**B**) Schematic of annealing assay (left). Non-denaturing gel showing RAD52 RNA-DNA annealing (top-middle) and RAD52 DNA-DNA annealing (bottom-middle). Plot showing % of RNA-DNA and DNA-DNA annealing (right). Data shown as average ± SD, n=3. (**C**) Non-denaturing gel showing RAD52 RNA-DNA recombination in the presence of the indicated substrates with pre-annealed markers shown in right most lanes. (**D**) Non-denaturing gel showing RAD52-dependent RNA-DNA recombination (bridging) using nucleic acid substrates with different sequences than those used in Figure 2. (**E**) Electrophoresis mobility shift assay (EMSA) showing RPA binding to ssDNA (left) and pssDNA (right) substrates. (**F**) Plot showing RAD51 binding to fluorescein-conjugated RNA measured by fluorescence polarization. Data shown as average ± SD, n=3. (**G**) Non-denaturing gel showing RPA unwinding of DNA. * = ^{32}P label.

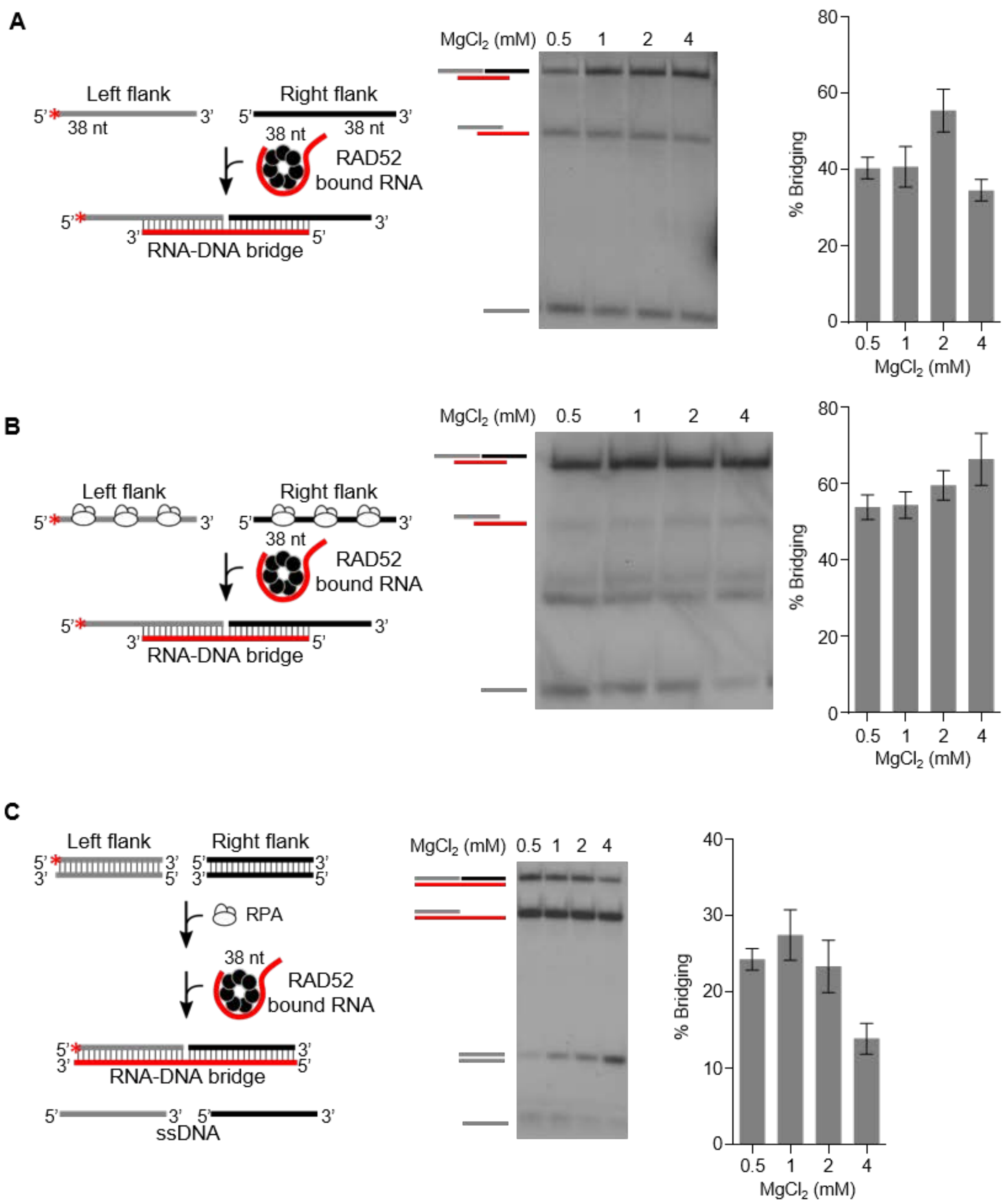

Supplementary Fig. 2 RAD52-dependent RNA-DNA recombination occurs under physiological concentrations of magnesium.

(**A**) Schematic of assay (left) Non-denaturing gel showing RAD52-dependent RNA-DNA recombination (bridging) of left and right homologous ssDNA flanks in the presence of the indicated amounts of $MgCl_2$ (middle). Graph showing % of RNA-DNA recombination (bridging) with the indicated amounts of $MgCl_2$ (right). Data shown as average + SD, n=3. (**B**) Schematic of assay (left) Non-denaturing gel showing RAD52-dependent RNA-DNA recombination (bridging) of left and right homologous ssDNA flanks pre-bound to RPA in the presence of the indicated amounts of $MgCl_2$ (middle). Graph showing % of RNA-DNA recombination (bridging) with the indicated amounts of $MgCl_2$ (right). Data shown as average + SD, n=3. (**C**) Schematic of assay (left) Non-denaturing gel

showing RAD52-dependent RNA-DNA recombination (bridging) of left and right homologous blunt-ended DNA flanks in the presence of the indicated amounts of $MgCl_2$ (middle). Graph showing % of RNA-DNA recombination (bridging) with the indicated amounts of $MgCl_2$ (right). Data shown as average ± SD, n=3. * = ^{32}P label.

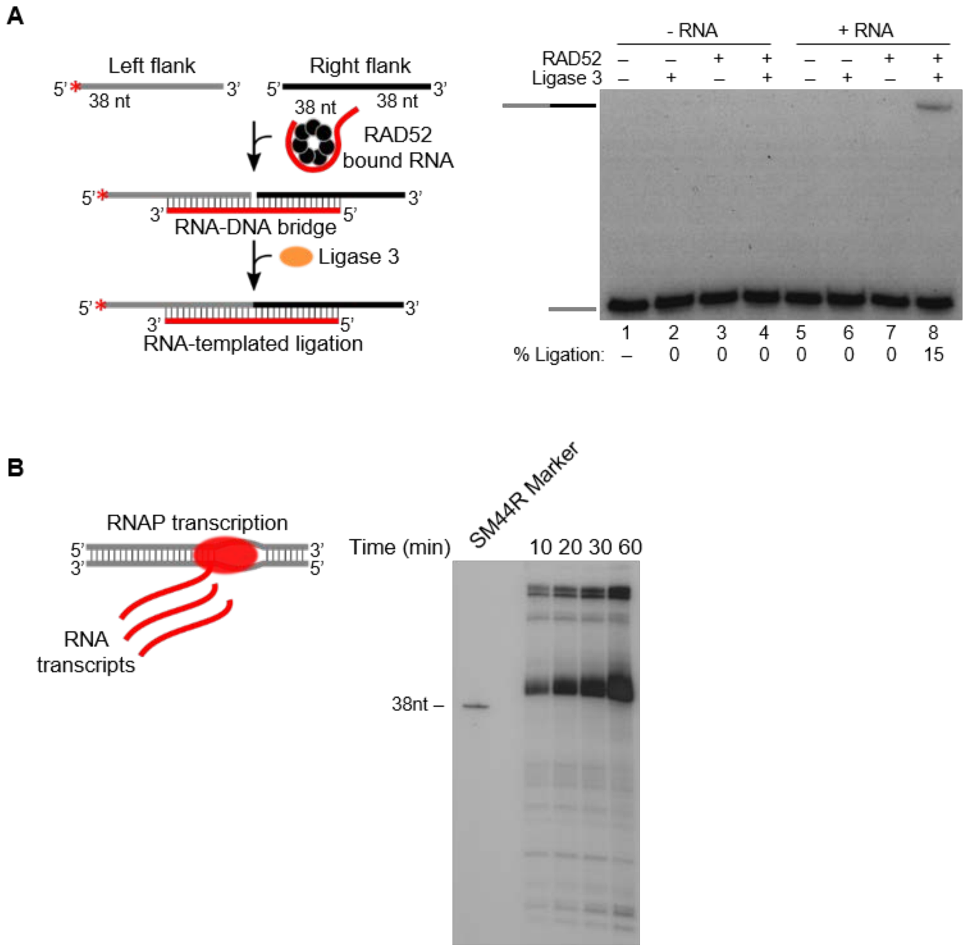

Supplementary Fig. 3 Additional controls for RAD52-dependent RNA-DNA recombinational repair.

(**A**) Schematic of assay (left). Denaturing gel showing RAD52 dependent RNA-DNA recombinational repair in the presence of the indicated proteins and RNA substrate (right). (**B**) Schematic of assay (left). Denaturing gel showing time course of transcription by T7 RNAP in the presence of [α-^{32}P]ATP (right). * = ^{32}P label.

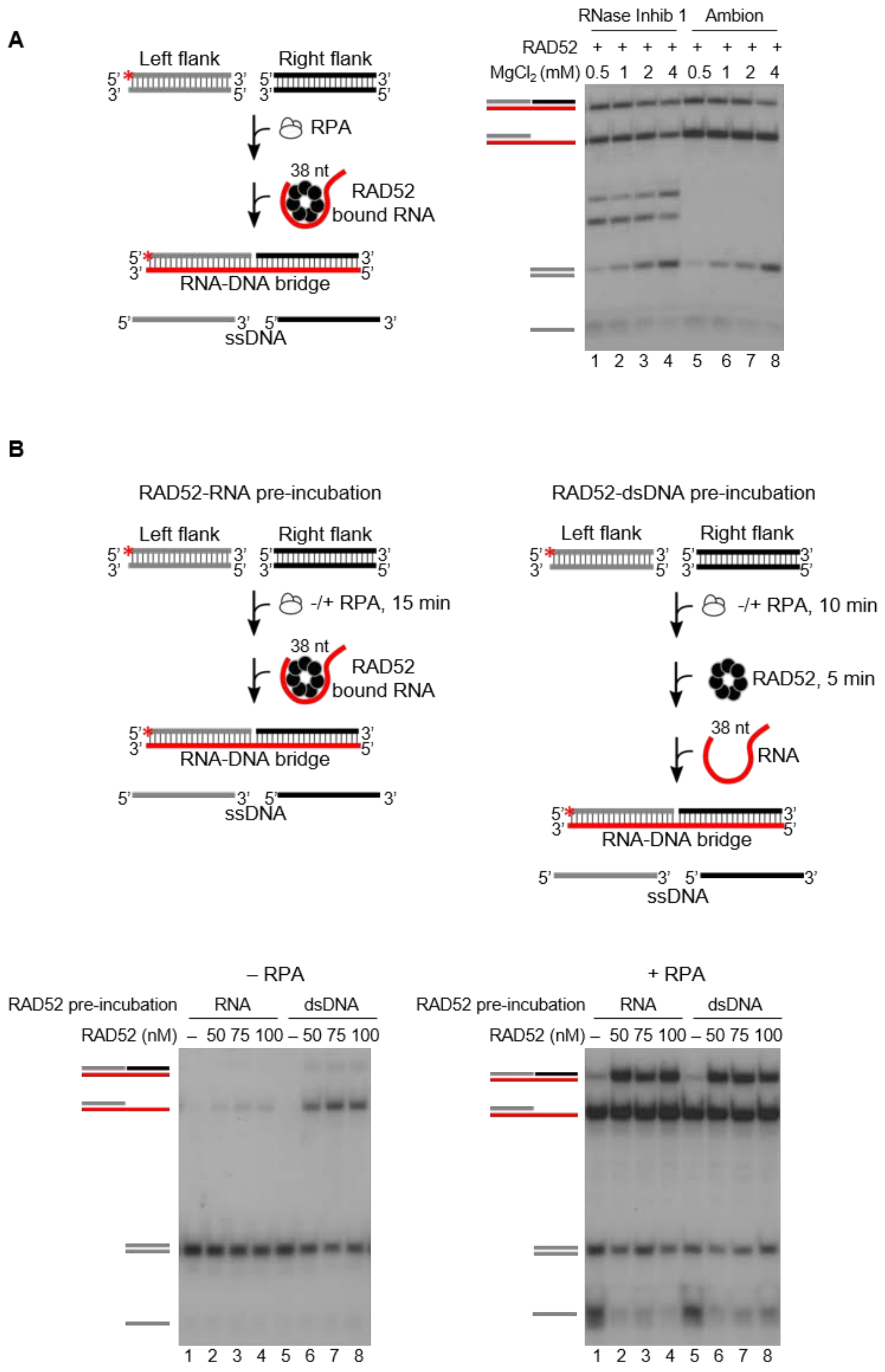

Supplementary Fig. 4 Controls for RAD52-dependent RNA-DNA recombination of blunt-ended DNA.

(**A**) Schematic of assay (left). Non-denaturing gel showing RAD52-dependent RNA-DNA recombination of blunt-ended DNA in the presence of the indicated RNase inhibitors (right). Disappearance of the intermediate molecular weight bands in lanes 5-8 indicates suppression of RNase contamination by the Ambion RNase inhibitor. (**B**) Schematic of assays employing either RAD52-RNA or RAD52-dsDNA pre-incubation steps in the

presence and absence of RPA (top). Non-denaturing gels showing RAD52-dependent RNA-DNA recombination employing the indicated pre-incubation steps in the presence (right panel) and absence (left panel) of RPA. Little or no difference in RNA-DNA recombination is observed in the presence of RPA (right panel). RAD52-dsDNA pre-incubation stimulates RNA-DNA half-bridge formation exclusively in the absence of RPA (left panel). These data are plotted as average $\pm$ SD in Figure 4C, n=3. * = ^{32}P label.

A

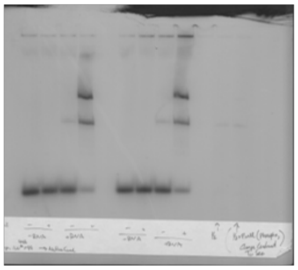

B

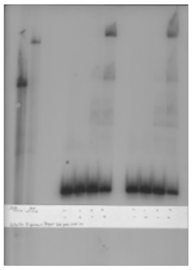

C

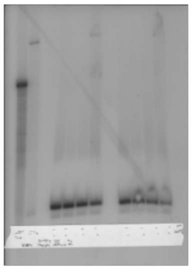

D

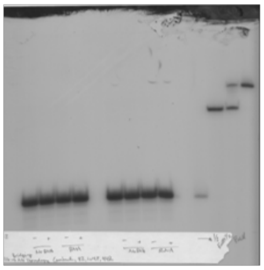

E

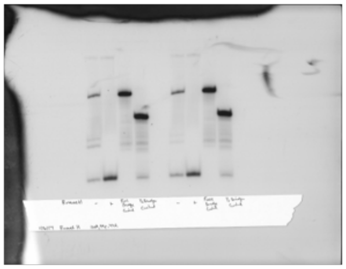

F

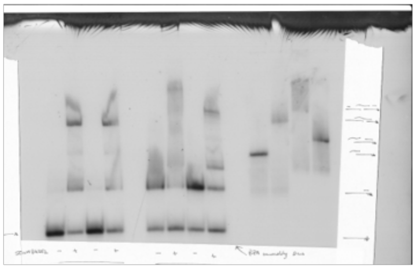

G

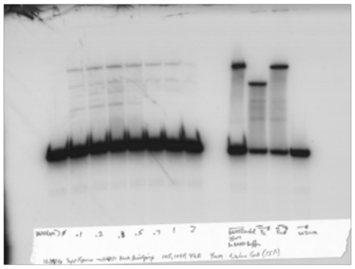

H

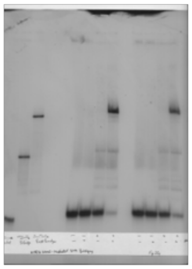

I

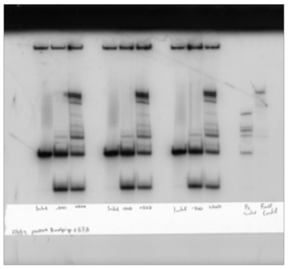

J

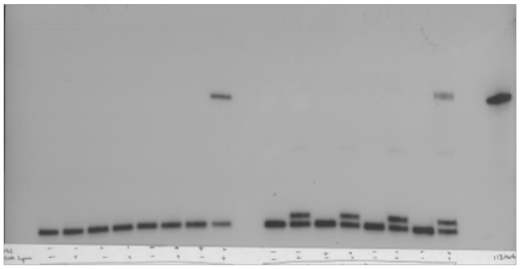

K

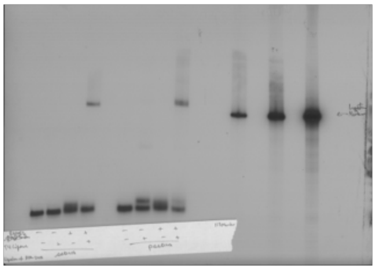

L

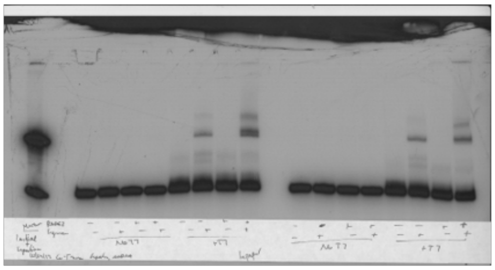

M

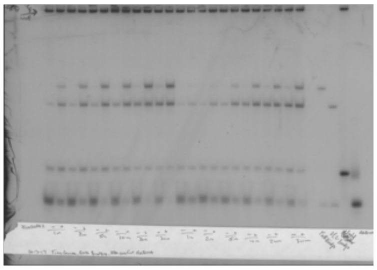

N

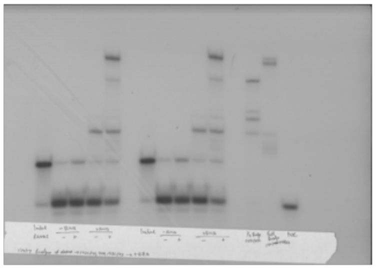

O

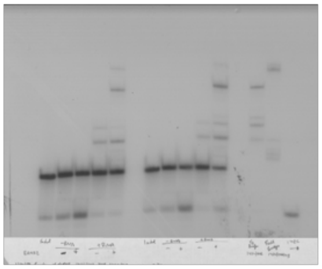

P

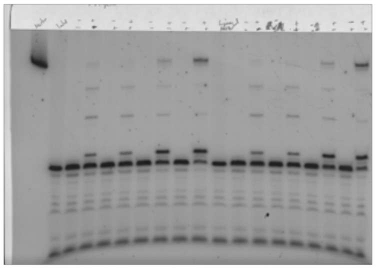

Q

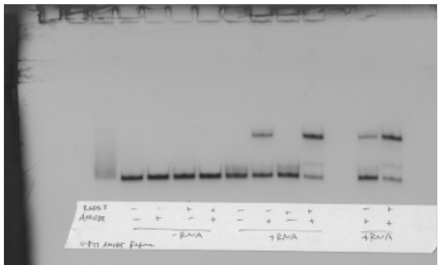

R

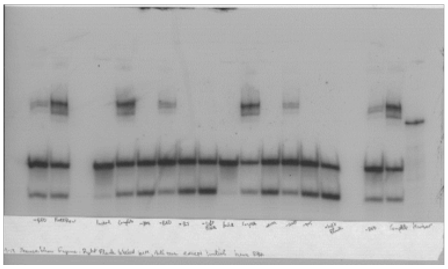

S

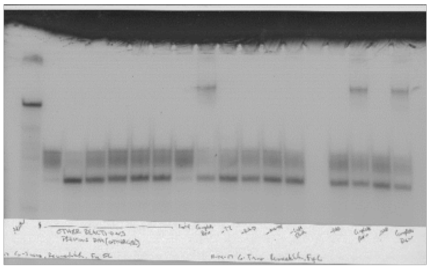

Supplementary Fig. 5 Uncropped gel images from main text.

(**A**) Fig 2A, 0 bp gap (**B**) Fig 2A, 6 bp gap (**C**) Fig 2A, 12 bp gap (**D**) Fig 2A, no homology (**E**) Fig 2B (**F**) Fig 2D (**G**) Fig 2F (**H**) Fig 2G (**I**) Fig 2H (**J**) Fig 2A (**K**) Fig 3B (**L**) Fig 3C (**M**) Fig 4A (**N**) Fig 4B, left (**O**) Fig 4B, right (**P**) Fig 4D (**Q**) Fig 5A (**R**) Fig 5B (**S**) Fig 5C

Supplementary Methods

Fluorescence Polarization Reactions were performed in 25 mM Tris-HCl pH 7.5, 1 mM DTT, 10 mM KCl, 0.01% NP-40, 0.1mg/ml BSA, 10% glycerol with 10 nM fluorescein-conjugated RNA (SM51R). Reactions containing RAD51 were supplemented with 2 mM $MgCl_2$, 4 mM $CaCl_2$, and 2 mM ATP. Reactions containing RAD52 were supplemented with 4 mM $MgCl_2$. After the addition of RAD51 or RAD52, reactions were incubated for 30 minutes at room temperature, then fluorescence polarization was determined using a Clariostar (BMG Labtech) plate reader (Figs. 1A, 1F).

RNA-DNA annealing Reactions were performed in 25 mM Tris-HCl pH 7.5, 1 mM DTT, 10 mM NaCl, 0.01% NP-40, 0.5 mM $MgCl_2$, 0.1 mg/ml BSA, 10% glycerol at 37° C. 30 nM RAD52 was pre-incubated with 4 nM RNA (SM44R) or ssDNA (SM44) for 5 min. Next, 4 nM 5'-^{32}P-radiolabeled complementary ssDNA (SM44C) was added and annealing occurred for 1 min. Reactions were terminated by the addition 0.4 μM cold ssDNA. Finally, reactions were treated with 2 μl of stop buffer (5 mg/ml proteinase K, 100 mM Tris-HCl pH 7.5, 1.5% SDS, 100 mM EDTA) for 15 min. Reactions were resolved in 15% non-denaturing polyacrylamide gels and visualized by autoradiography (Fig 1B).

RNA-dependent DNA recombination of ssDNA and pssDNA Figs 1C, 1D: Reactions were performed in 20 μl volume of buffer A (25 mM Tris-HCl pH 7.5, 1 mM DTT, 10 mM NaCl, 0.01% NP-40, 0.1 mg/ml BSA, 10% glycerol) at 37° C. Reactions contained 0.5 mM $MgCl_2$. 30 nM RAD52 was pre-incubated with 4 nM RNA (SM46R) for 5 min. Next, equimolar concentrations of indicated 5'-^{32}P-radiolabeled 4 nM ssDNA (SM104P and SM105) were added. Reactions were terminated after 1 min or indicated times by the addition 0.4 μM cold ssDNA, then treated with 2 μl of stop buffer (5 mg/ml proteinase K, 100 mM Tris-HCl pH 7.5, 1.5% SDS, 100

mM EDTA) for 15 min. Nucleic acids were resolved in 15% non-denaturing polyacrylamide gels and visualized by autoradiography.

RPA EMSA In the case of RPA binding to ssDNA or pssDNA (Fig. 1E), 20 nM RPA was incubated with 8 nM ssDNA or pssDNA for 15 min in 25 mM Tris-HCl pH 7.5, 1 mM DTT, 10 mM NaCl, 0.01% NP-40, 0.5 mM $MgCl_2$, 0.1 mg/ml BSA, 10% glycerol at 37° C. Reactions were resolved in 5% non-denaturing polyacrylamide gels and visualized by autoradiography. In the case of RPA unwinding of dsDNA (Fig. 1G), RPA was incubated with 83 bp dsDNA (SM132/SM133) for 15 min in 25 mM Tris-HCl pH 7.5, 1 mM DTT, 10 mM NaCl, 0.01% NP-40, 0.5 mM $MgCl_2$, 0.1 mg/ml BSA, 10% glycerol at 37° C. Reactions were terminated by the addition 0.4 μM cold ssDNA and treated with 2 μl of stop buffer (5 mg/ml proteinase K, 100 mM Tris-HCl pH 7.5, 1.5% SDS, 100 mM EDTA) for 15 min. Reactions were resolved in 15% non-denaturing polyacrylamide gels and visualized by autoradiography.

Magnesium titration during ssDNA and dsDNA bridging: Figs 2A, 2B, 2C: Reactions were performed as described in the main text (See methods for main text Figs. 2A, 2D, 4A).

RNA-DNA ligation involving human Ligase 3 RAD52-dependent RNA bridging reactions were performed in 20 μl of buffer A as described above (main text Fig. 2A), followed by ligation with 50 nM human ligase 3. During ligation, $MgCl_2$ was supplemented to 4 mM $MgCl_2$ (Fig. 3A) for 2 hr at 25 °C. Reactions were supplemented with 1 mM ATP during ligation. Reactions were terminated by the addition of stop buffer (45% formamide, 20 mM EDTA). Reactions were resolved in 12% denaturing (urea) polyacrylamide gels and visualized by autoradiography.

In vitro transcription Transcription was performed in 20 mM Tris-HCl pH 8.0, 1 mM DTT, 6 mM $MgCl_2$, and 20 units RNase inhibitor (Ambion) for 1 hr. Reactions contained 50 nM T7 RNA polymerase and 50 nM transcription template (SM85/SM85C), 400 μM rGTP, rCTP, rUTP, and 100 μM rATP. Reactions were visualized by incorporation of [α-^{32}P] rATP. Reactions were terminated by stop buffer (45% formamide, 20 mM EDTA), resolved in 20% denaturing (urea) polyacrylamide gels, and visualized by autoradiography.

RNA-dependent DNA recombination of dsDNA In the case of testing different RNase inhibitors (Fig. 4A), reactions were performed as described in the main text (see main text Fig. 4A) containing 2 mM $MgCl_2$ and 20 units RNase inhibitor (Ambion) or 20 units of RNase inhibitor I (Invitrogen SUPERase-In). In the case of testing RAD52 pre-incubation (Fig. 4B), see methods for main text Figure 4C.
