# Supplementary material for: How RNA transcripts coordinate DNA recombination and repair
Source: Nat Commun. 2018 Mar 15;9:1091. doi: 10.1038/s41467-018-03483-7 (PMC5854605; doi:10.1038/s41467-018-03483-7)
Supplement: Supplementary file 2 — Peer Review File [file 41467_2018_3483_MOESM2_ESM.pdf]

PEER REVIEW FILE

<u>Reviewers' comments</u>:

Reviewer #1 (Remarks to the Author):

Genetic studies in yeast suggest that RNA transcripts facilitate the repair of DNA double-strand breaks, particularly when RNA:DNA hybrids are stabilized by eliminating RNase H activity. RNA-directed repair is Rad52 dependent and independent of Rad51. Since Rad52 is known to anneal complementary single-stranded DNAs (ssDNA), or RNA and ssDNA (Keskin et al, 2014), it seems likely to directly participate in RNA-directed repair. In this study, the authors propose two alternative mechanisms for RNA-directed repair – RNA bridging and RNA-templated repair - and present biochemical evidence using reconstitution assays to support both models. While the data are generally supportive of the models, the substrates for the ligation assay seem somewhat contrived and most of the assays lack quantitation, particularly those involving in vitro transcription.

The bridging mechanism would require overhangs with different polarity on each side of the break to support ligation, contrary to our current knowledge of end resection. Thus, substrates shown in Fig 2, with the exception of part G, are not really physiologically relevant. However, the RPA-mediated "melting" of the dsDNA substrates used in Fig 2G, would mean that the reaction is really no different to the assays with ssDNA. The reaction shown in Fig 2G is similar to the "reverse strand exchange" assay recently demonstrated to be promoted by Rad52 (Mazina et al, 2017, Mol Cell). In light of results from Mazina et al, it is surprising that so little product is seen in the assay with Rad52 in the absence of RPA (Fig S1H). Perhaps the difference is whether Rad52 is bound to the dsDNA or RNA. Although the Mazina et al paper was published after this manuscript was submitted, the authors do need to consider and discuss their data in light of this new study. It is interesting to note that in the Mazina et al study no gels were shown for the Rad52+RPA assays and they may not have considered the contribution of the RPA dsDNA "melting" activity to product formation.

The yeast genetic studies showed greatly decreased efficiency of repair when reverse transcriptase (RT) activity was reduced, interpreted as cDNA generated from the transcribed RNA being used as a repair donor. It would be useful to compare the efficiency of bridging using ssDNA and RNA donors.

Although the % bridging is presented in Fig 2, there is no quantitation in the other figures. This is especially important for Figs 3C and 4C, where the ligation and RNA-templated repair

products are minor and even detected in the absence of Rad52. The mean values from at least 3 independent assays should be shown with standard deviation.

Minor comments:
Line 33: Sequence dependent implies recognition of a specific sequence by Rad52.
Fig. 2G: What is the product in lanes 4 and 5 that migrates slower than dsDNA left flank? Data from Fig S1H should be in the main figure.
Fig 4B. There are many unlabeled products on the gel shown. It is very hard to understand this assay without knowing what all the new products are. It is not obvious to me what the major product is in the assay lacking RT if there is no homology between the left and right flanks.

Reviewer #2 (Remarks to the Author):

The authors described that RAD52 directly cooperates with RNA as a sequence-dependent ribonucleoprotein complex to promote two related modes of RNA-DNA repair. Consistent with the existing literature that RNA transcript can act as the template for DNA repair through recombination, the authors demonstrate the role of RAD52 in assembling recombinant RNA-DNA hybrids to facilitate the ligation of homologous DNA breaks, using an artificial in vitro system. The information about the role of RAD52 in coordinating homology-directed DNA recombination supports the previous studies, however the authors claim about the report of DNA DSB repair in the absence of a DNA donor is not correct as it has been already reported several times in the literature. Therefore, the information reported in paper does not qualify for publication in Nature Communication.

Reviewer #3 (Remarks to the Author):

The results in Figure 2 are interesting, but puzzling, at least when compared to RAD52-mediated annealing of ssDNA. It has been published that with DNA oligonucleotides, the rate of spontaneous annealing of the protein-free ssDNA displays the fastest rate of annealing. RPA blocks this spontaneous annealing, as seen for RNA-annealing, and RAD52 reverses this inhibition by mediating annealing to the RPA-ssDNA; again, this is seen for the RNA-annealing reaction. However, for DNA, RAD52-mediated annealing is still slower than the rate of annealing of the free ssDNA (protein-devoid), but this is not what the authors observe for RAD52-medaited annealing of RNA. In fact, inexplicably, the authors do not observe spontaneous annealing of the RNA, which seems impossible (assuming there is no stable secondary structure in these RNAs). This could be because the kinetic order of these reactions is

different (first- vs. second-order). Consequently, it is important that the authors examine this apparent discrepancy by measuring the annealing rate using at least three different concentration of RNA oligos. This is an essential experiment because, on the face of it, the reaction does not mimic the canonical DNA-annealing reaction.

The use of a low magnesium ion concentration (0.5 mM) for the experiments in Figure 2 (though, somewhat curiously, not in subsequent experiments) is also a problem. The magnesium concentration used, 0.5 mM, is not physiological, which is typically considered to be ~ 3 mM (a range of 1-5 mM is regarded as appropriate). As the authors point out, RPA artifactually denatures duplex DNA at 0.5 mM. Hence, in some reactions, the DNA and RNA substrates might be actually being denatured by the RPA. Also, given comment (1) above, the use of the low [Mg2+] might explain the discrepancy with the DNA annealing reactions. Hence, the authors must reexamine these reactions at higher concentrations of Mg2+, e.g., 3 mM and the 6 mM concentration apparent used later in the paper. These experiments must be done, as they bear on the likely physiological significance of these results. In addition, it is recommended that the kinetic order of RNA-annealing, +/- RAD52 with and without RPA, be examined at the higher, more relevant Mg-concentrations.

Responses to Reviewers' comments:

Reviewer #1 (Remarks to the Author):

Genetic studies in yeast suggest that RNA transcripts facilitate the repair of DNA double-strand breaks, particularly when RNA:DNA hybrids are stabilized by eliminating RNase H activity. RNA-directed repair is Rad52 dependent and independent of Rad51. Since Rad52 is known to anneal complementary single-stranded DNAs (ssDNA), or RNA and ssDNA (Keskin et al, 2014), it seems likely to directly participate in RNA-directed repair. In this study, the authors propose two alternative mechanisms for RNA-directed repair – RNA bridging and RNA-templated repair - and present biochemical evidence using reconstitution assays to support both models. While the data are generally supportive of the models, the substrates for the ligation assay seem somewhat contrived and most of the assays lack quantitation, particularly those involving in vitro transcription.

Authors' response:
We very much thank the reviewer for their fair critique and we agree with their comments and suggestions. We have therefore majorly revised the report to fully accommodate the referee's concerns. For example, we agree that the original data are supportive of the models, and that the substrates for the ligation assay seem somewhat contrived. We also agree that some of the original data lacked quantitation, such as those involving in vitro transcription. We provide a point by point response below.

Reviewer #1 (Remarks to the Author):
The bridging mechanism would require overhangs with different polarity on each side of the break to support ligation, contrary to our current knowledge of end resection. Thus, substrates shown in Fig 2, with the exception of part G, are not really physiologically relevant. However, the RPA-mediated "melting" of the dsDNA substrates used in Fig 2G, would mean that the reaction is really no different to the assays with ssDNA.

Authors' response:
We agree that the substrates shown in original Fig. 2 (except for those in original Fig. 2G) may not be physiologically relevant. These substrates were simply used to incrementally investigate the underlying mechanism of RAD52-RNA dependent DNA repair. For example, we first evaluated this process using ssDNA left and right flanking molecules that are bridged together by RAD52 and recombinant RNA which allows for ligation. We incrementally move towards dsDNA substrates by then evaluating this process using partially ssDNA substrates (pssDNA). After fully understanding the basic mechanisms, we move on to more relevant substrates such as blunt ended DNA that accurately models DSBs. We emphasize that since this is a newly discovered process, we feel it is beneficial for the reader to understand the underlying mechanisms in a simplified form before moving on to the more complex reactions involving blunt ended DNA. For example, in the revised manuscript we fully explore the various conditions and rates, as well as the necessary magnesium concentrations for the many simplified RNA-mediated repair reactions using ssDNA and pssDNA before moving on to the more complex RNA-mediated DSB repair reactions.

In the new revised manuscript, we dedicate an entire figure to RNA-mediated repair of blunt ended DNA which represents the most physiologically relevant substrate (see new Fig. 4; new Supplementary Fig. 4; model of mechanism illustrated in Fig. 1A). We now show that this process occurs efficiently, is dependent on RNA, RPA and RAD52, and is easily reproducible as shown in triplicate and quantitated (see new Fig. 4 and new Supplementary Fig. 4 controls). For example, Fig. 4A demonstrates a time course of RNA-dependent bridging of a homologous DSB with blunt ends in the presence and absence of RAD52. Fig. 4B shows this reaction requires RPA, RAD52 and homologous RNA. New supplementary Fig. 2C shows that this mechanism occurs under various physiological concentrations of magnesium, and Fig. 4C and Supplementary Fig. 4B analyze the effects of pre-incubation conditions for RAD52 in this reaction. We additionally show that this reaction stimulates DNA synapses that are sealed by ligase, resulting in actual DSB repair in new Fig. 4D. Taken together, the addition of the data in Fig. 4 and Supplementary Fig. 4 and Supplementary Fig. 2C fully elucidate this new physiologically relevant mechanism of RAD52-RNA dependent DSB repair.

Reviewer #1 (Remarks to the Author):

The reaction shown in Fig 2G is similar to the “reverse strand exchange” assay recently demonstrated to be promoted by Rad52 (Mazina et al, 2017, Mol Cell). In light of results from Mazina et al, it is surprising that so little product is seen in the assay with Rad52 in the absence of RPA (Fig S1H). Perhaps the difference is whether Rad52 is bound to the dsDNA or RNA. Although the Mazina et al paper was published after this manuscript was submitted, the authors do need to consider and discuss their data in light of this new study.

Authors’ response:
We agree with the reviewer about addressing the potential involvement of RAD52 reverse strand exchange in light of our findings. We therefore fully evaluated whether RAD52 reverse strand exchange is involved in our system. For example, since our reactions involving blunt ended DNA are the closest to those used in the recent reverse strand exchange study, we examined whether pre-incubating RAD52 with the dsDNA rather than with RNA affected the outcome or efficiency of our reactions in which RAD52 promotes RNA-dependent bridging of a DSB resulting in a synapsis of the DSB (see new Fig. 4C). The reactions were performed as shown in 4C diagrams; RAD52 was either pre-incubated with dsDNA or with RNA. Another variable included the presence or absence of RPA since we show that it necessary for RNA bridging of a DSB (see new Fig. 4C and new Fig. 4B). The data demonstrate that pre-incubation of RAD52 with dsDNA or RNA has little or no effect on RNA bridging of a DSB (new Fig. 4C and Supplementary Fig. 4B, bottom). These data also confirm a necessary role for RPA in RAD52-RNA bridging of a homologous DSB (new Fig. 4C). We note that reverse strand exchange does seem to stimulate RNA-DNA half-bridge formation (i.e. a single RNA-DNA hybrid at one of the DNA flanks), but this occurs only in the absence of RPA (Supplementary Fig. 4B, bottom left). We now fully address reverse strand exchange in our newly revised manuscript in the text and Figures (Fig. 4C and Supplementary Fig. 4B), and these data conclusively show that reverse strand exchange does not stimulate RNA bridging of a DSB, but does stimulate half-bridge formation exclusively in the absence of RPA. We emphasize that RPA is essential for our RNA-bridging of a DSB reactions.

Reviewer #1 (Remarks to the Author):
It is interesting to note that in the Mazina et al study no gels were shown for the Rad52+RPA assays and they may not have considered the contribution of the RPA dsDNA “melting” activity to product formation.

Authors’ response:
We agree with the reviewers insight and also are surprised the Mazina et al study did not show any gels. Nevertheless, RPA unwinding of dsDNA has been well documented, and this is referenced in our paper and shown in Supplementary Fig. 1G and in Figures 2H and 4A and Fig. 4B, left.

Reviewer #1 (Remarks to the Author):
The yeast genetic studies showed greatly decreased efficiency of repair when reverse transcriptase (RT) activity was reduced, interpreted as cDNA generated from the transcribed RNA being used as a repair donor. It would be useful to compare the efficiency of bridging using ssDNA and RNA donors.

Authors’ response:
We agree with the reviewer, and have not thoroughly compared our bridging reactions using ssDNA versus RNA. This is now compared and quantitated in new Fig. 2C (without RPA) and Fig. 2E (with RPA). In both cases, the rate of bridging of the left and right flanking DNA is slightly faster with ssDNA versus RNA. However, the yield of products is slightly higher with RNA. In the presence of RPA there is no spontaneous bridging in the absence of RAD52 regardless of whether RNA or ssDNA is used (Fig. 2E). Minor spontaneous bridging is observed in the absence of RPA and with ssDNA (Fig. 2C).

Reviewer #1 (Remarks to the Author):
Although the % bridging is presented in Fig 2, there is no quantitation in the other figures. This is especially important for Figs 3C and 4C, where the ligation and RNA-templated repair products are minor and even detected in the absence of Rad52. The mean values from at least 3 independent assays should be shown with standard deviation.

Authors’ response:
We agree with the reviewer that the major assays should be performed in triplicate and shown as the mean with standard deviation for a more rigorous biochemical study. Fig. 3C was further optimized and now is

represented as three independent assays shown as the mean +/- SD with and without RAD52, and with and without RNA polymerase (Fig. 3C, right). We believe the appearance of slightly extended DNA (i.e. double-bands and extended bands) exclusively in the presence of RNAP in Fig. 3C is due to its ability to slightly extend DNA using NTPs (i.e. terminal transferase activity).

Reviewer #1 (Remarks to the Author):
Minor comments:
Line 33: Sequence dependent implies recognition of a specific sequence by Rad52.

Authors' response:
We have now changed this terminology as "sequence-directed" or "homology-directed" in all cases

Reviewer #1 (Remarks to the Author):
Fig. 2G: What is the product in lanes 4 and 5 that migrates slower than dsDNA left flank? Data from Fig S1H should be in the main figure.

Authors' response:
During the revision we found that the addition of a RNase inhibitor in our reactions substantially reduced these intermediate molecular weight byproducts, indicating that these products are due to RNase contamination in our reactions. This is now explained in the main text and shown as Supplementary Fig. 4A. The majority of the revised experiments were performed with a RNase inhibitor such as those in Fig. 4A, Supplementary Fig. 4A and 4B, Supplementary Fig. 2C, Fig. 5, and Fig. 3C to suppress these occasional intermediate molecular byproducts formed by degradation of the RNA.

Original Fig. S1H is now included in the main Fig. 4B, right.

Reviewer #1 (Remarks to the Author):
Fig 4B. There are many unlabeled products on the gel shown. It is very hard to understand this assay without knowing what all the new products are. It is not obvious to me what the major product is in the assay lacking RT if there is no homology between the left and right flanks.

Authors' response:
We again thank the reviewer for their insight and detailed critique. We have further optimized this reaction since there were obvious byproducts. The reactions are now performed with a RNase inhibitor which substantially reduces many byproducts that are due to partial RNAse degradation of the RNA. We also further optimized the assay to the extent where the desired product is now the major band Fig. 5B, lane 2. This recombination product is also quantitated (+/- SD) in the presence and absence of RAD52 as shown in the right plot (new Fig. 5B, right). The original Fig. 4C transcript based assay was also further optimized and quantitated, and does not result in multiple byproducts (new Fig. 5C). Only a single byproduct is observed (the lower ssDNA product due to RPA unwinding)(new Fig. 5C).

Reviewer #2 (Remarks to the Author):

The authors described that RAD52 directly cooperates with RNA as a sequence-dependent ribonucleoprotein complex to promote two related modes of RNA-DNA repair. Consistent with the existing literature that RNA transcript can act as the template for DNA repair through recombination, the authors demonstrate the role of RAD52 in assembling recombinant RNA-DNA hybrids to facilitate the ligation of homologous DNA breaks, using an artificial in vitro system. The information about the role of RAD52 in coordinating homology-directed DNA recombination supports the previous studies, however the authors claim about the report of DNA DSB repair in the absence of a DNA donor is not correct as it has been already reported several times in the

literature. Therefore, the information reported in paper does not qualify for publication in Nature Communication.

Authors' response:
Although the reviewer does not favor publication of our manuscript in Nature Communications based on our findings, we note our previous correspondence with the editor (Stephane Larochelle) who is in favor publishing the article in the event that we satisfy the other reviewers with a majorly revised manuscript.

Reviewer #3 (Remarks to the Author):
The results in Figure 2 are interesting, but puzzling, at least when compared to RAD52-mediated annealing of ssDNA. It has been published that with DNA oligonucleotides, the rate of spontaneous annealing of the protein-free ssDNA displays the fastest rate of annealing. RPA blocks this spontaneous annealing, as seen for RNA-annealing, and RAD52 reverses this inhibition by mediating annealing to the RPA-ssDNA; again, this is seen for the RNA-annealing reaction. However, for DNA, RAD52-mediated annealing is still slower than the rate of annealing of the free ssDNA (protein-devoid), but this is not what the authors observe for RAD52-medaited annealing of RNA. In fact, inexplicably, the authors do not observe spontaneous annealing of the RNA, which seems impossible (assuming there is no stable secondary structure in these RNAs). This could be because the kinetic order of these reactions is different (first- vs. second-order). Consequently, it is important that the authors examine this apparent discrepancy by measuring the annealing rate using at least three different concentration of RNA oligos. This is an essential experiment because, on the face of it, the reaction does not mimic the canonical DNA-annealing reaction.

Authors' response:
We thank the reviewer for their insight into RAD52 biochemistry and thorough and fair critique. With all due respect, we believe there may have been a misinterpretation on the part of the referee. For example, although the referee claims that, "for DNA, RAD52-mediated annealing is still slower than the rate of annealing of the free ssDNA (protein-devoid), but this is not what the authors observe for RAD52-medaited annealing of RNA", multiple labs have shown that RAD52 increases the rate of annealing between complementary free ssDNA (protein-devoid), and this has also been shown to be the case for RAD52 stimulating the rate of annealing between RNA and ssDNA. Some of these references showing RAD52 increasing the rate of annealing for protein-devoid ssDNA (and RNA) are listed below.

RAD52 stimulation of the rate of annealing in the absence of RPA has previously been demonstrated by several labs (including our own) and these peer reviewed reports are referenced below:

1. Tyrosine phosphorylation enhances RAD52-mediated annealing by modulating its DNA binding. Honda M, Okuno Y, Yoo J, Ha T, Spies M. EMBO J. 2011 Jul 29;30(16):3368-82. doi: 10.1038/emboj.2011.238.EMBO PMID: 15175261

2. Rothenberg E, Grimme JM, Spies M, Ha T. Human Rad52-mediated homology search and annealing occurs by continuous interactions between overlapping nucleoprotein complexes. Proc Natl Acad Sci U S A. 2008 Dec 23;105(51):20274-9. doi: 10.1073/pnas.0810317106. Epub 2008 Dec 11.

3. Human Rad52 binds and wraps single-stranded DNA and mediates annealing via two hRad52–ssDNA complexes. Grimme JM, Honda M, Wright R, et al. Human Rad52 binds and wraps single-stranded DNA and mediates annealing via two hRad52–ssDNA complexes. Nucleic Acids Research. 2010;38(9):2917-2930. doi:10.1093/nar/gkp1249.

4. Correlation of biochemical properties with the oligomeric state of human rad52 protein. Lloyd JA, Forget AL, Knight KL. J Biol Chem. 2002 Nov 29;277(48):46172-8. Epub 2002 Sep 10.

5. DNA strand annealing is promoted by the yeast Rad52 protein. Mortensen UH, Bendixen C, Sunjevaric I, Rothstein R. Proc Natl Acad Sci U S A. 1996 Oct 1;93(20):10729-34. PMID: 8855248

6. Human Rad52 protein promotes single-strand DNA annealing followed by branch migration. Reddy G, Golub EI, Radding CM. Mutat Res. 1997 Jun 9;377(1):53-9. PMID: 9219578

7. DNA annealing mediated by Rad52 and Rad59 proteins. Wu Y, Sugiyama T, Kowalczykowski SC. J Biol Chem. 2006 Jun 2;281(22):15441-9. Epub 2006 Mar 25.

8. Small-Molecule Disruption of RAD52 Rings as a Mechanism for Precision Medicine in BRCA-Deficient Cancers. Chandramouly G, McDevitt S, Sullivan K, Kent T, Luz A, Glickman JF, Andrake M, Skorski T, Pomerantz RT. Chem Biol. 2015 Nov 19;22(11):1491-1504. doi: 10.1016/j.chembiol.2015.10.003. Epub 2015 Nov 5.

9. Protein dynamics during presynaptic-complex assembly on individual single-stranded DNA molecules. Gibb B, Ye LF, Kwon Y, Niu H, Sung P, Greene EC. Nat Struct Mol Biol. 2014 Oct;21(10):893-900. doi: 10.1038/nsmb.2886. Epub 2014 Sep 7.PMID: 25195049

10. Molecular pathways: understanding the role of Rad52 in homologous recombination for therapeutic advancement. Lok BH, Powell SN. Clin Cancer Res. 2012 Dec 1;18(23):6400-6. doi: 10.1158/1078-0432.CCR-11-3150. Epub 2012 Oct 15. Review. PMID: 23071261

11. Reappearance from Obscurity: Mammalian Rad52 in Homologous Recombination. Hanamshet K, Mazina OM, Mazin AV. Genes (Basel). 2016 Sep 14;7(9). pii: E63. doi: 10.3390/genes7090063. Review. PMID: 27649245

12. Role of the Rad52 amino-terminal DNA binding activity in DNA strand capture in homologous recombination. Shi I, Hallwyl SC, Seong C, Mortensen U, Rothstein R, Sung P. J Biol Chem. 2009 Nov 27;284(48):33275-84. doi: 10.1074/jbc.M109.057752. Epub 2009 Oct 6. PMID: 19812039

13. Transcript-RNA-templated DNA recombination and repair. Keskin H, Shen Y, Huang F, Patel M, Yang T, Ashley K, Mazin AV, Storici F. Nature. 2014 Nov 20;515(7527):436-9. doi: 10.1038/nature13682. Epub 2014 Sep 3.

Thus, we are somewhat confused by the reviewers statement below:

"However, for DNA, RAD52-mediated annealing is still slower than the rate of annealing of the free ssDNA (protein-devoid), but this is not what the authors observe for RAD52-medaited annealing of RNA. In fact, inexplicably, the authors do not observe spontaneous annealing of the RNA, which seems impossible (assuming there is no stable secondary structure in these RNAs)."

Despite this possible misunderstanding that RAD52 does not increase the rate of annealing in the absence of RPA (references above show otherwise), we have now thoroughly examined DNA-DNA and RNA-DNA spontaneous annealing and RAD52 mediated annealing rates of these substrates in the presence and absence of RPA using our bridging assays in the revised manuscript and find that the rates of these reactions are very similar regardless of whether RNA or DNA is used. We also would like to emphasize that the tri-molecular annealing reactions involving RNA-mediated annealing of two flanking homologous DNAs has not been previously investigated (see schematics in Fig. 2A and Fig. 2D). For example, we now show rates of these reactions involving either RNA- or DNA- mediated annealing (bridging) of two flanking homologous ssDNA substrates with and without RAD52 in new Fig. 2C and new Fig. 2E. Only Bimolecular annealing between DNA-DNA or RNA-DNA by RAD52 has been investigated. Therefore the rates and spontaneous annealing of our reactions are likely different than those previously described for bimolecular annealing. We have also performed simple bimolecular DNA-DNA and RNA-DNA annealing by RAD52 in Supplementary Fig. 1B, as shown in previous studies. We note that although both RNA-DNA and DNA-DNA can spontaneously

anneal in these reactions, previous studies and our data (Supplementary Fig. 1B) show that RAD52 facilitates this process making the rate of annealing in the absence of RPA faster than that observed for spontaneous annealing. We also emphasize that the reaction time interval of the simple RNA-DNA and DNA-DNA bimolecular annealing reactions in Supplementary Fig. 1B is 1 min. We also note that in the bimolecular annealing reaction between RNA and DNA in Supplementary Figure 1B, we do observe some spontaneous annealing between RNA and DNA in the absence of RAD52 during the short time course of 1 min. DNA-DNA spontaneous annealing occurs more readily at the same time point in the absence of RAD52 likely due to less secondary structure compared to RNA.

We again point out that the majority of reactions throughout the main text exclusively examine RNA- or DNA-mediated annealing (bridging) of two flanking homologous ssDNA substrates. Thus, this form of trimolecular annealing (i.e. annealing between 3 strands of nucleic acid) has not previously been investigated. Various models of this (including ssDNA, pssDNA and dsDNA) are illustrated in the following new Figures: Fig. 1A; Fig. 2A,D,G,H; Fig. 3; Fig. 4.

We note that in response to the reviewers statement,"In fact, inexplicably, the authors do not observe spontaneous annealing of the RNA, which seems impossible (assuming there is no stable secondary structure in these RNAs).", we do observe some spontaneous annealing of the three separate nucleic acid molecules (i.e. bridging; trimolecular annealing) in the new revised Fig. 2C. This shows that ssDNA can spontaneously anneal to two flanking ssDNA molecules at a slow rate. Again we note that this form of trimolecular annealing has not previously been described. For RNA, there is much less spontaneous annealing between it and the two flanking homologous ssDNA substrates in the same figure likely due to more prominent secondary structure. Considering that our reactions involve 3 molecules instead of 2 molecules in the annealing process, our reactions are more complex which can explain the low rate of spontaneous annealing between 3 nucleic acid substrates, regardless of whether DNA or RNA are used. It is well known that DNA-DNA and RNA-DNA in bimolecular annealing reactions, that these substrates can spontaneously anneal over time. However, the ability of 3 homologous nucleic acid substrates to anneal (i.e. trimolecular annealing, bridging) has never been investigated.

We note also that we now thoroughly analyzed the different rates of trimolecular annealing (bridging) reactions under various conditions (+/-RAD52, +/-RPA, RNA versus ssDNA as a bridging molecule) in new Fig. 2C and Fig. 2E. These data demonstrate that DNA-mediated bridging with RAD52 is only slightly faster than RNA-mediated bridging with RAD52 (Fig. 2C). We observe a similar slight increase in the rate of trimolecular annealing in the presence of RPA and RAD52 when ssDNA is used as a bridging molecule versus RNA (new Fig. 2E). Hence, these data show that the rates of trimolecular annealing between RNA-DNA-DNA and DNA-DNA-DNA are very similar, indicating that the kinetic order of these reactions are the same. We also show that the bimolecular annealing rate between RNA-DNA and DNA-DNA in the presence of RAD52 is identical (see new Supplementary Fig. 1a, right), again indicating that the kinetic order of these more simple annealing reactions is the same. We believe these data now thoroughly differentiate between the different reaction rates and we have also better explained these novel forms of bridging (trimolecular annealing) reactions in the main text to make it more clear that these assays examine a novel form of annealing involving 3 nucleic acid substrates as follows:

"This process would require RAD52 to assemble a RNA-DNA hybrid that spans both ends of the DNA break, resulting in a RNA-DNA recombinant bridge (Fig. 1A). This tri-molecular form of RAD52 annealing, referred to herein as bridging, has not previously been investigated."

If the reviewer believes we should further investigate the kinetic order of the RNA-DNA-DNA vs DNA-DNA-DNA trimolecular reactions in the presence of RAD52 despite their similar reaction rates, we are prepared to do so, but would need an extension for the 3 month period allowed for manuscript revision.

Reviewer #3 (Remarks to the Author):
The use of a low magnesium ion concentration (0.5 mM) for the experiments in Figure 2 (though, somewhat curiously, not in subsequent experiments) is also a problem. The magnesium concentration used, 0.5 mM, is

not physiological, which is typically considered to be ~ 3 mM (a range of 1-5 mM is regarded as appropriate). As the authors point out, RPA artifactually denatures duplex DNA at 0.5 mM. Hence, in some reactions, the DNA and RNA substrates might be actually being denatured by the RPA. Also, given comment (1) above, the use of the low [Mg2+] might explain the discrepancy with the DNA annealing reactions. Hence, the authors must reexamine these reactions at higher concentrations of Mg^{2+}, e.g., 3 mM and the 6 mM concentration apparent used later in the paper. These experiments must be done, as they bear on the likely physiological significance of these results. In addition, it is recommended that the kinetic order of RNA-annealing, +/- RAD52 with and without RPA, be examined at the higher, more relevant Mg-concentrations.

Authors' response:
We again thank the reviewer for their thorough and fair review. We agree that the effects of different magnesium concentrations should be assessed regarding the physiological relevance of our reactions. We have now thoroughly tested multiple magnesium concentrations within physiological range for several different major reactions in our study. For example, we now have dedicated an entire Supplementary figure 2 to this analysis. We now show that all of the major RNA-mediated bridging reactions involving RAD52 occur at physiological range (1-4 mM magnesium), and in most cases the concentration of magnesium has no significant effect. Thus, our data clearly support this form of bridging reactions at physiological concentrations of magnesium as a range of 1-5 mM is regarded as appropriate as stated by the reviewer. The original manuscript overlooked this important analysis. The use of higher magnesium in the original ligation based reactions later in the paper was simply due to convenience since the ligase is reported to work best at this concentration. The use of higher magnesium (i.e. 6 mM) in new Fig. 3C and Fig. 5 is to accommodate the optimal conditions of RNA polymerase and reverse transcriptase. However, we have already shown throughout the manuscript, especially in Supplementary Fig. 2 that magnesium concentrations of 1-4 mM work well for all RNA-DNA bridging reactions. In addition to the thorough analysis of magnesium concentration on the effects of our bridging reactions, we list the magnesium concentrations used for the new data in the revised manuscript below:

Fig. 2C, 2E: 2 mM $MgCl_2$
Supplementary Fig. 2: 1-4 mM $MgCl_2$ titrated
Fig. 3C: 6 mM $MgCl_2$
Supplementary Fig. 3B: 6 mM $MgCl_2$
Fig. 4A-C: 2 mM $MgCl_2$
Fig. 4D: 6 mM $MgCl_2$
Fig. 5: 6 mM $Mgcl_2$
Supplementary Fig. 4A, 4B: 2 mM $MgCl_2$

Lastly, we also thoroughly analyzed the rate of our tri-molecular annealing (bridging) reactions using more physiologically relevant Mg concentration of 2 mM as shown in Fig. 2C and Fig. 2E as suggested by the reviewer. We hope the reviewer will now agree that our study carefully uses physiologically relevant concentrations of Mg and thoroughly analyzes the rates of our complex trimolecular annealing reactions with RNA versus DNA as well as other variables (+/-RAD52, +/-RPA), and clearly explains this new form of tri-molecular annealing in the text to better distinguish it from previously described bimolecular annealing between DNA-DNA or RNA-DNA.
